# Unraveling the Mechanical Behavior of Softwood Secondary Cell Walls through Atomistic Simulations

**DOI:** 10.1021/acs.biomac.4c01806

**Published:** 2025-05-27

**Authors:** Lucas N. Trentin, Amadeus C. S. Alcântara, Carlos G. T. Batista, Munir S. Skaf

**Affiliations:** † Institute of Chemistry, University of Campinas, Campinas, SP 13084-862, Brazil; ‡ Center for Computing in Engineering & Sciences (CCES), University of Campinas, Campinas, SP 13083-861, Brazil; § Department of Computational Mechanics, School of Mechanical Engineering, University of Campinas, Campinas, SP 13083-860, Brazil

## Abstract

The plant cell wall (PCW) is a remarkable biomaterial, endowing plants with strength, stiffness, and defense against pathogens and chemical agents. This complex structure, mainly composed of cellulose in a matrix of hemicellulose, lignin, and water, exhibits impressive mechanical properties. However, the link between its molecular architecture and macroscopic mechanics is not fully understood. This study uses molecular dynamics simulations to examine the nanomechanical behavior of spruce wood’s S2 layer. Multicomponent models including cellulose, hemicellulose (xylan and mannan), lignin, and water were developed. Simulations showed that water acts as a “molecular lubricant”, mediating critical interactions between the components of the system. Tension and compression tests on the models displayed realistic mechanical behavior. Our results show that cellulose microfibrils bear the primary load, while lignin dissipates stress under compression. These findings offer new insights into the relationship between the molecular structure and mechanical function in this complex biomaterial.

## Graphical abstract

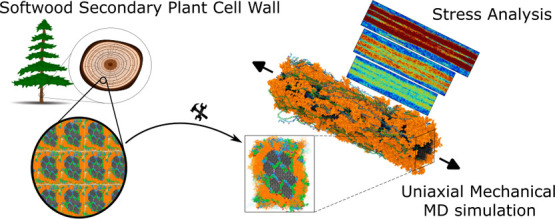

## Introduction

The plant cell wall (PCW) is a complex biomolecular structure crucial for plant growth, development, and mechanical stability,^1^ primarily composed of lignocellulosic biopolymers, including cellulose, hemicellulose, and lignin. PCW also incorporates ions, proteins, and minor organic compounds.^2^ This intricate structure provides vital support and chemical protection against physical stresses and pathogens.

The PCW is typically subdivided into the middle lamella (ML), primary cell wall (CW1), and up to three secondary cell wall layers (S1, S2, and S3).^3^ Among these layers, the secondary cell wall, particularly rich in cellulose, plays a critical role in providing high mechanical stiffness and strength to plants. This is particularly evident in tall plants such as spruce, pine, oak, and birch, where the secondary cell wall can constitute up to 90% of the thickness of the PCW.^4^ The S2 layer, which comprises approximately 80% of the thickness of the secondary cell wall, is indispensable to ensure robust mechanical properties.^5^ Its unique architecture, resembling reinforced concrete, features cellulose fibrils embedded within a matrix of hemicellulose, lignin, and water.

Plant species exhibit unique variations in the composition, spatial distribution, and interactions of cellulose, hemicellulose, and lignin within their cell walls, leading to distinct material properties. For example, while grasses and herbs exhibit a lignin content comparable to hardwoods (17–24 and 18–25%, respectively),^6–8^ their lignin composition and lignin-cellulose interactions differ significantly from those of hardwoods.^9^ Softwoods are characterized by a higher abundance of mannan compared to xylan and possess highly hydrated lignin.^9,10^ In contrast, hardwoods exhibit extensive lignin–lignin assemblies, increasing the hydrophobicity of the cell wall and indirectly promoting the association of 3-fold xylans with cellulose fibrils.^11^ These distinct structural features at the molecular level ultimately underpin the characteristic mechanical properties of softwoods (lightweight and flexible) and hardwoods (high density and stiffness). Figure 1 illustrates a schematic representation of the multiscale structure of a softwood.

**Figure 1:**
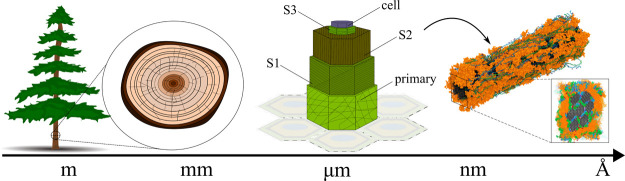
Schematic illustrates the multiscale structure of a softwood. On the left, a softwood tree is depicted, with a zoomed-in cross-sectional view of its trunk. At the microscale level, individual plant cells are shown, each surrounded by cell walls composed of primary (CW1) and secondary wall layers (S1, S2, and S3). The S2 layer, the focus of our atomistic model, is further depicted as a composite material consisting of cellulose (black), hemicelluloses (green and blue), and lignin (orange).

Hemicelluloses play a vital role in mediating lignin-cellulose interactions and even influencing fibril–fibril contacts. The carbohydrate decoration pattern significantly impacts these interactions, as demonstrated by Berglund and colleagues.^12^ They observed that mannan acts as an adhesive in bacterial cellulose, while xylan exhibits lubricating properties, reducing fibril adhesion. Furthermore, Penttilä et al.^13^ highlighted the role of hemicelluloses in influencing fibril packing and inducing a transition in cellulose from type Iα to Iβ, with xylan chains amplifying these effects. The even spacing of xylan decorations, while allowing for minor branching patterns, enables these hemicelluloses to interact effectively with both the hydrophilic and hydrophobic surfaces of cellulose.^14–16^

In addition to hemicellulose, the distribution of water significantly influences the structural properties of the plant cell wall.^17,18^ The water content plays a key role in determining the mechanical behavior of the cell wall. For instance, living, hydrated wood exhibits elasticity and recovers after deformation, whereas dried wood often fractures in a brittle manner.^19,20^ Moreover, confined water within the cellulose microfibrils promotes fibril–fibril interactions.^21^ However, thermal pretreatments reduce the water content within these microfibrils, leading to fibril coalescence, increased cellulose crystallinity, and altered stress distribution.^22,23^ Interestingly, dried softwoods can partially regain interfibril cellulose distances after rewetting.^24^ Nevertheless, some cellulose chains, previously in direct contact with water, become bound to hemicellulose, hindering subsequent water reentry.^18^ While water does not rehydrate the xylan-cellulose interface, it fully restores mannan-cellulose contacts, with a small fraction of mannan chains remaining mobile.^18^ Despite lignin’s inherent tendency toward self-aggregation, which increases hydrophobicity,^11^ softwood lignins exhibit a more dispersed distribution compared to their hardwood counterparts. This dispersion facilitates closer proximity to highly hydrated regions and even enables water entrapment within the lignin matrix.

Another intriguing aspect of PCWs is the mechanical implication of the covalent bonds between lignin and hemicellulose within the lignin-carbohydrate complexes (LCCs). Busse-Wicher et al.^14^ demonstrated that acetyl groups attached to xylans can enhance noncovalent interactions between lignin and hemicellulose. Lignin monomers are assembled within the PCW via a radical mechanism,^25,26^ enabling cross-linking with hemicelluloses and bundling of carbohydrate polymers.^25^ These covalent linkages have been shown to contribute significantly to the mechanical strength of the S2 layer in bamboo microfibrils.^27^ Experimental evidence indicates that lignin within LCCs constitutes a substantial portion of the total lignin mass, typically ranging from 90 to 95%.^28,29^ These LCCs consist mainly of multiple small lignin fragments linked to single xylan, mannan, and cellulose chains, suggesting a relatively low degree of cross-linking. Notably, 92% of covalently bound lignin is associated with xylan and mannan, while only 8% is linked to cellulose.^28^ Furthermore, in complexes with hemicelluloses, lignin can constitute 30–40% of the total mass, while in complexes with cellulose, this proportion decreases to approximately 15%.^30^

Despite significant advances in our understanding of the structural and chemical properties of PCWs, our knowledge of their nanoscale mechanical behavior and the underlying molecular mechanisms remains limited.^31^ Molecular dynamics (MD) simulations offer a powerful tool for investigating the nanoscale properties of these complex systems by capturing the cell wall at the atomistic level.^32^ Previous MD studies have focused mainly on small-scale models, providing valuable information on specific interactions within the PCW, such as cellulose-hemicellulose-lignin interactions,^14,33^ the role of calcium ions as mediators of xylan–xylan interactions,^34^ the importance of water in maintaining softwood secondary plant cell walls,^18,21,35,36^ and the mechanical properties of individual components.^27,36–38^ However, these studies have largely relied on simplified systems, hindering our ability to accurately predict the macroscopic behavior of larger fragments of PCWs. While recent studies have employed coarse-grained models to investigate higher scale phenomena, such as the relationship between cellulose organization and cell wall extensibility in onion epidermal (primary) cell walls,^39^ and atomistic models have been developed for hardwood systems, specifically for Populus S2 layers, based on solid-state NMR data,^40,41^ a comprehensive and experimentally validated atomistic model for softwood S2 layers is currently lacking. One notable exception is the recent work of Penttilä and collaborators,^42^ who investigated the effects of moisture content and degree of lignification on the interactions between cellulose microfibrils in secondary PCW of spruce using MD simulations of a molecular model of the cell wall nanostructure and X-ray scattering data.

In this study, we investigate the mechanical behavior of the PCW’s S2 layer using MD simulations to model tensile loading conditions. The MD simulations presented here incorporate key aspects of the biochemistry of these biocomposites by considering fundamental physical principles and the input of experimental data. This approach can provide insights into the nanoscale chemical and mechanical behavior of biological materials. Here, we employ computational techniques to explore the molecular mechanisms underlying the stiffness and strength of the S2 layer. To this end, we develop a multicomponent S2 model based on experimental data from spruce (*Picea abies*) softwood. We then consider two variations of this model: one representing a dried cell wall and another lacking covalent bonds between hemicellulose and lignin. Simulations of these models provide information about how the cellulose microfibrils and the lignin-polysaccharide matrix interact under strain. Furthermore, our analysis of stress distributions and hydration offers new insight into the mechanical role of each component within the S2 layer of softwoods.

## Methods

### Atomistic Spruce S2 Model

Our spruce S2 model incorporates cellulose, hemicelluloses, and lignins, in addition to water and counterions. To accurately represent the chemical composition, we utilized the comprehensive study by Martínez-Abad et al.,^10^ which quantified the major constituents in a spruce wood sample. Notably, Martínez-Abad et al. employed a sequential hydrothermal treatment with subcritical water to isolate hemicellulose populations while preserving their delicate decorations. This approach, in contrast to conventional acid or base extractions that often lead to significant depolymerization, ensured the integrity of the hemicellulose structures. Consequently, the data from Martínez-Abad et al. served as an invaluable source of experimental parameters for the development of our S2 model.

Figure 2 depicts the mass percentage of each biopolymer within the simulated S2 layer. Cellulose microfibrils were generated using Cellulose-Builder,^43^ while polydispersed lignin fragments were constructed with LigninBuilder.^44^ Hemicellulose and lignin-carbohydrate complexes (LCCs) were modeled by modifying single cellulose chains within the VMD psfgen plugin.^45^ The molecular system was assembled using Packmol,^46^ enabling the controlled packing of individual molecules and constituents within the simulation box. Subsequent molecular dynamics simulations, encompassing minimization, equilibration, and production runs, were conducted using NAMD^47^ versions 2.14 and 3.0, employing the CHARMM36 force field parameters^48–53^ and the TIP3P water model.^54^ Three distinct model variations were evaluated: a fully hydrated system (‘wet’), a dehydrated system (‘dry’), and a system lacking LCCs (‘nolcc’).

**Figure 2:**
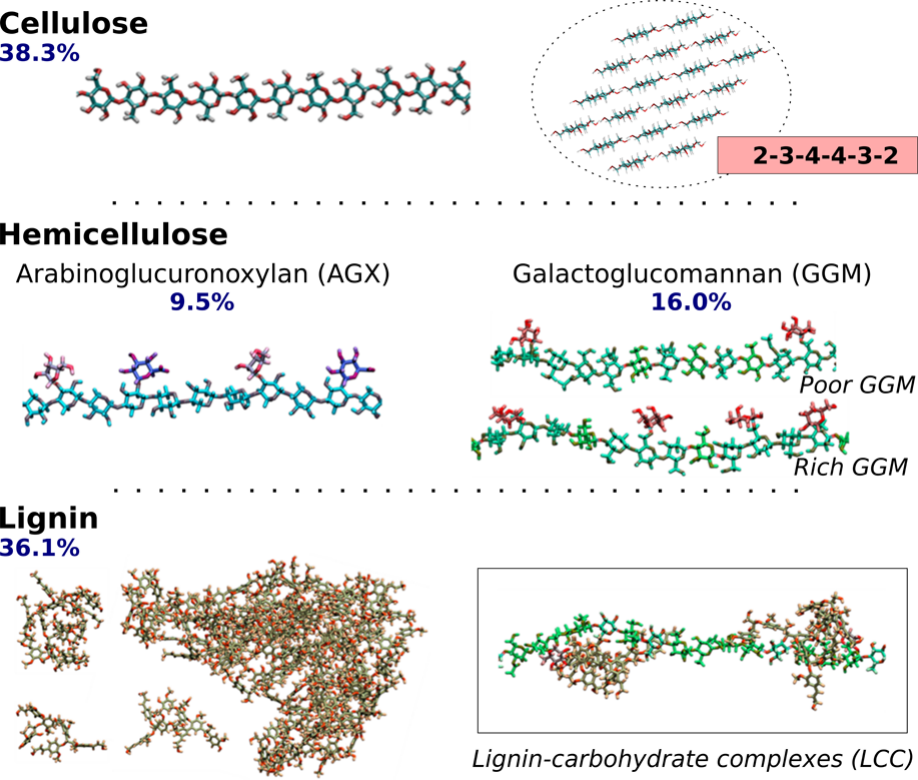
Structural features and composition of our softwood secondary PCW model. The following color scheme is used to represent the different monosaccharide and lignin units: glucose in cellulose (gray), xylose (cyan), arabinose (pink), glucuronic acid (purple), mannose (sea green), glucose in mannans (lime green), galactose (red), guaiacyl, and p-hydroxyphenyl units in lignin (ochre). Percentages shown in the figure represent the mass fraction of each constituent within the model.

#### Cellulose Microfibril Habit

In this work, we employed the 18-chain Iβ cellulose^55–58^ microfibril model, consisting of 100 β-d-glucose units linked by β-(1 → 4) glycosidic bonds and arranged in a 2–3–4–4–3–2 habit.^18,59,60^ Seven microfibrils are bundled together in a honeycomb-patterned cluster, with each cellulose chain covalently connecting the first and last residues across periodic boundary conditions along the major axis.

#### Hemicellulose Composition

Each hemicellulose chain contains 96 sugar residues. Thirty arabinoglucuronoxylan (AGX) chains were included, each featuring a xylose backbone with evenly spaced branches of glucuronic acid (GlcA) and arabinose (Ara). The GlcA:Ara ratio was set to 17:11,^10,16,61,62^ excluding 4-O-methylation on GlcA units, which was deemed to have a minimal impact on the overall model architecture. Forty-five acetylated galactoglucomannan (GGM) chains were incorporated. All GGM chains contain a backbone of mannose (Man) and glucose (Glc) monomers with α-galactose (Gal) decorations. Gal-rich GGMs (30 chains) exhibit an acetylation degree of 0.4 and a Man:Glc:Gal ratio of 4:1:0.3, while Gal-poor GGMs (15 chains) exhibit an acetylation degree of 0.1 and a Man:Glc:Gal ratio of 3:1:1.2.^10^ Like in α-(1 → 6)­Gal substitutions, O-acetylations occur on the O2 or the O3 positions of Man units only. Throughout this work, GGMs and AGXs are interchangeably referred to as mannans and xylans.

#### Lignin Composition and Lignin-Carbohydrate Complexes

Three hundred polydispersed lignin fragments were generated with degrees of polymerization (DP) ranging from 6 to 249, based on experimental data for spruce.^44,63^ These globular lignin structures predominantly consist of guaiacyl (G) and p-hydroxyphenyl (H) units in a 19:1 ratio, with a branching degree of 0.3.^44^ Approximately 90% of the total lignin mass is complexed with xylans and mannans, forming lignin-carbohydrate complexes (LCCs) where lignin constitutes approximately 30–40% of the complex mass. LCCs typically exhibit roughly seven hemicellulose-lignin linkages per 100 aromatic monomers, primarily through phenyl-glycoside and benzyl-ether bonds.^30,64^ To mitigate potential biases arising from the high solubility of low-DP LCCs,^65^ lignin fractions with DP values below 10, corresponding to an expected lignin mass fraction of approximately 10%, were modeled to form only noncovalent bonds with carbohydrates, facilitating their dispersion within the matrix. Hemicellulose chains were associated with an average of 3–4 midsized lignin structures (average molar mass: 2773 g/mol) to achieve the desired hemicellulose-lignin ratios within the LCCs, considering the molar masses of the hemicellulose molecules: 16,985 g/mol (xylan), 18,138 g/mol (mannan poor), and 20,668 g/mol (mannan rich). Conversely, the “nolcc” model, by definition, lacks any covalent bonds between lignin and carbohydrates.

#### Water Content and Counterions

Softwoods typically exhibit a hydration water content ranging from 30 to 60% of their total mass, depending on the wood section (heartwood or sapwood).^66^ However, these values represent the overall water content of the wood, which includes water present within the vascular system. Cell wall water content in gymnosperms can vary significantly, ranging from 5 to 40% of the total dry mass, and may be strongly dependent on the ambient humidity.^67,68^ This water plays an essential role in determining the overall cell wall architecture. In our model, an intermediate water content of 23% of the total polymeric mass is incorporated. To maintain electroneutrality within the system, counterions were necessary. We included about 0.2% of calcium ions (Ca^2+^) in the model, as they are the most common cation in softwoods and influence xylan interactions.^34^ Acetylated mannans and GlcA branches within xylan (p*K*_*a*_ ≈ 3) are predominantly deprotonated at physiological pH for spruce wood (pH ≈ 5).^69^

#### Model Assembling Protocol

The initial model comprised a macrofibril within a rectangular simulation box in which a central cellulose microfibril was surrounded by six identical microfibrils, forming a honeycomb-like arrangement.^70^ AGX and GGM chains were incorporated to mediate interactions between the microfibrils.^71–73^ A schematic representation of the building protocol is provided in Figure S1. Calcium ions were included to neutralize the charge arising from the presence of acetate groups within the hemicellulose structures. The simulation box was initially filled with a subsaturated amount of water molecules, anticipating that water condensation on the microfibril surfaces would drive them toward the experimentally observed separation distances.^35^ A minimum distance of 15 Å was maintained between periodic images of the microfibrils (Figure S1A). Following this initial assembly, 40 ns of dynamics at 300 K were conducted to allow hemicellulose chains to adhere to the microfibrils, where hemicellulose adopt a predominantly 2-fold screw conformation^74^ (Figure S1B).

Larger lignin fragments were positioned and oriented on the carbohydrate-rich surface of the microfibril cluster to favor potential lignin-carbohydrate interactions. This step aimed to simulate the initial stages of the covalent bond formation between lignin and hemicellulose within the extrafibrillar matrix. Additional water molecules were introduced into the simulation box, and the system was equilibrated for an additional 5 ns until the lignin structures condensed and stabilized (see Figure S1C). Subsequently, five hemicellulose chains were covalently cross-linked to lignin molecules from the previously created lignin-carbohydrate complexes (LCCs).

The remaining constituents, including additional LCC constructs, free small oligolignin fragments, hydration water, and counterions, were distributed around the core structure to achieve a composition consistent with experimental measurements. To incorporate the chemical diversity of LCCs, ten distinct types of complexes were constructed, each comprising different combinations of xylan, mannan, and medium-sized lignin fragments. These complexes were pre-equilibrated in water for 5 ns to ensure that the lignin fragments and their linkages did not adopt unrealistic conformations in a densely populated model. In all of the steps described up to this point, no structural restrictions were employed in order to allow defects to be naturally inserted into the crystalline structure of cellulose.

Subsequently, a series of minimization and equilibration steps were employed to bring the system to a compact structure, with the external pressure gradually increasing to a maximum of 3000 bar until the system stabilized its density. At a temperature of 300 K, large lignin fragments may lack sufficient thermal energy to explore their conformational space and fill gaps in the simulation box, which would prevent the system from reaching an adequate density. Therefore, our building protocol included fast heating steps to ensure an even distribution of matrix polymeric chains, as done in the construction of similar models in the literature.^37^ Additionally, the high pressures required to compact the constituents into a dense structure can induce stress accumulation due to the rise of internal forces, which heating helps to dissipate. Therefore, the backbone of the cellulose chains was constrained during pressurization and heating steps to maintain the crystalline structure of microfibrils (Figure S1D).

The aforementioned minimization and equilibration protocol consists of four main steps (summarized in Table S1). Initially, the steepest descent minimization (1000 steps) was followed by a 200 ps NPT simulation at 300 K and 1 bar to equilibrate the water molecules. Next, sequential NPT simulations (50 ps each) were performed with a gradual increase in pressure (Stage 21 in Table S1) to densify the system. To enhance the conformational flexibility of matrix hemicellulose and lignin, alternating NPT pressurization (300 K, 2 fs time step) and NVT heating (800 K, 1 fs time step) steps were conducted until the system density stabilized (Stage 42). During these stages, harmonic restraints (force constant: 2 kcal mol^–1^ Å^–2^) were applied to the C1, C2, C3, C4, C5, and O5 atoms of the cellulose backbone to preserve the crystalline structure.^34,75–77^ In the final stage, the system was gradually returned to the desired conditions of 300 K and 1 bar through a 700 ps simulation, during which the applied pressure was gradually released, and the constraints on the cellulose backbone were removed.

The described protocol resulted in the creation of a “wet” model. Two additional model variations were generated from this base model: a “dry” model with reduced water content and a ‘nolcc’ model lacking covalent bonds between hemicellulose and lignin. The “dry” model was generated by reducing the water content to 1% of the polymeric mass. In contrast, the “nolcc” model was created from the “wet” model by removing all covalent linkages between hemicellulose and lignin within the existing LCCs. The “wet” and “nolcc” models comprised approximately 900,000 atoms, whereas the “dry” model contained ∼700,000 atoms.

#### Molecular Dynamics Simulations

Molecular dynamics simulations were conducted for at least 750 ns, reaching 1 μs for both ‘dried’ and ‘nolcc’ models, with a 2 fs time step in the NPT ensemble. We used the Langevin thermostat^78^ and barostat^79^ to regulate the temperature (300 K) and pressure (1 bar). The Particle Mesh Ewald (PME) method was applied to handle the long-range electrostatic interactions with an on-grid spacing of 1 Å.^80^ Short-ranged van der Waals and real-space components of the electrostatic interactions were truncated at a cutoff radius of 12 Å, with smooth switching set at 10 Å.

### Assessing Mechanical Properties of PCW Models

#### Tensile and Compressive Tests

To assess the mechanical properties of the models, we employed tensile and compressive tests simulated using the Large-scale Atomic/Molecular Massively Parallel Simulator (LAMMPS),^81^ a classical MD code with a focus on material science applications. Prior to LAMMPS simulations, the final configuration of each model after the 750 ns or 1 μs NAMD runs was converted into LAMMPS data files using the *charmm2lammps.pl* conversion tool. This ensured compatibility with LAMMPS software. To verify the integrity of the data conversion, a brief relaxation simulation was performed in LAMMPS, confirming no significant changes in the system behavior. Subsequently, tensile and compressive tests were conducted using the parameters outlined in Table 1.

**Table 1:** Parameters of the Mechanical Stress Simulations in LAMMPS

parameter	value
run time	30 ps (fast)/1 ns (slow)
time step	2 fs
axial direction	*z*
engineering strain rate	1 × 10^–5^ ps (fast)/1 ×10^–7^ ps (slow)
ensemble	NPT
temperature	310 K
pressure	1.0 atm
inner pairwise cutoff (LJ + Coulombic)	10.0 Å
outer pairwise cutoff (LJ + Coulombic)	12.0 Å
neighbor skin (extra distance cutoff)	1.0 Å
particle–particle particle-mesh accuracy	1 × 10^–4^
thermostat algorithm	Langevin dynamics
barostat algorithm (on *xy*-plane)	Nosé-Hoover

Two sets of simulations were performed, differing primarily in their strain rates: “fast” simulations with a higher engineering strain rate (|ε̇_*zz*_| = 10^–5^ fs^–1^) and “slow” simulations with a lower strain rate (|ε̇_*zz*_| = 10^–7^ fs^–1^). The higher strain rate in the “fast” simulations facilitated the observation of dynamic intra- and intermolecular interactions during rapid loading. Conversely, the lower strain rate in the “slow” simulations was employed to obtain more accurate estimates of Young’s modulus, which can be influenced by high strain rates. A positive strain rate value indicates elongation of the simulation box along the *z*-axis, while a negative value signifies compression. Periodic boundary conditions were maintained throughout the simulations, including the continuity of covalent bonds across the periodic boundaries. The NPT ensemble was applied in the x-plane to allow for adjustments in the box dimensions during axial deformation. The LAMMPS fix deform command was utilized to implement both tensile and compressive deformations by systematically altering the box dimensions along the *z*-axis (eq 1).

$$L_{z}\left(t\right)=L_{z}\left(t_{0}\right)\left[{\mathrm{\varepsilon ̇}}_{zz}\left(t\right)\cdot \left(t-t_{0}\right)+1\right]$$ 1

The initial box length *L*_*z*_(*t*_0_) is deformed over time into *L*_*z*_(*t*) by a strain rate ε̇_*zz*_(*t*) along the *z*-axis. During each step of the deformation, the symmetric pressure tensor is computed by using eq 2:

$$P_{ij}=\frac{1}{V}\overset{N}{\underset{k=1}{\sum }}m_{k}\nu_{ik}\nu_{jk}+\frac{1}{V}\overset{N^{\prime }}{\underset{k=1}{\sum }}r_{ik}f_{jk}$$ 2

The pressure tensor *P*_*ij*_ is decomposed into the kinetic (left) and virial (right) tensors, where *i* and *j* represent the Cartesian coordinates, *V* is the volume of the simulation box, *N* is the total number of atoms within the primary simulation domain, *N’* includes atoms from neighboring subdomains (for periodic boundary conditions), *m*_*k*_ is the mass of the k-th atom, *r*_*ik*_ and *v*_*ik*_ are the *i*th component of the position and velocity, respectively, of the k-th atom, and *f*_*jk*_ is the j-th component of the resultant force on the k-th atom.

#### Young’s Modulus and Per-Atom Stresses

Young’s Modulus (*E*) for each model was determined by assuming linear elastic behavior, consistent with Hooke’s Law. The per-atom pressure, −*P*_*zz*_, was considered to be the negative of the per-atom stress (σ_*zz*_ = −*P*_*zz*_ = *E*ε_*zz*_). For each simulation, a stress–strain curve was generated. The elastic region of this curve was then fitted with a linear regression using the least-squares method. The slope of this fitted line directly corresponds to Young’s Modulus.

It is important to note that the presented methodology represents one approach for assessing mechanical properties from MD simulations. Alternative methods, such as free energy calculations,^82^ can provide more thermodynamically rigorous estimations. However, due to the significant computational cost associated with calculating the potential of mean force for systems of this size, these methods were deemed computationally prohibitive for the current study.

Finally, per-atom stress values were computed during the tensile and compressive tests. While significant variations in per-atom stress values are expected and may not be suitable for quantitative analysis, their spatial distribution provides valuable insights into stress propagation within the plant cell wall model. This spatial analysis aids in identifying potential failure locations and the constituents most susceptible to mechanical stress. The output trajectory files and per-atom quantities generated by these simulations were analyzed using OVITO.^83^ Further details regarding the mechanical testing protocols and simulation methodologies in other natural fibers can be found elsewhere.^84,85^

## Results and Discussion

### Atomistic Spruce S2 Models

Following the construction protocol, each system (wet, dry, and nolcc) underwent extensive simulation runs to ensure stability (see Figure S2). The equilibration protocol required multiple refinements to overcome challenges encountered during the early simulation stages. These challenges included: excessive constraint values, rapid pressure increases leading to system disruption, distortions in the crystalline microfibril structure due to insufficient or absent constraints during high-pressure stages, and inadequate system densities resulting from insufficient pressurization or an insufficient number of heating steps prior to pressurization. The final equilibration protocol yielded a stable “wet” model (Figure 3) with a density of 1.32 g cm^–3^, consistent with experimental and theoretical values for plant cell walls (1.3–1.5 g cm^–3^).^37,86^

**Figure 3:**
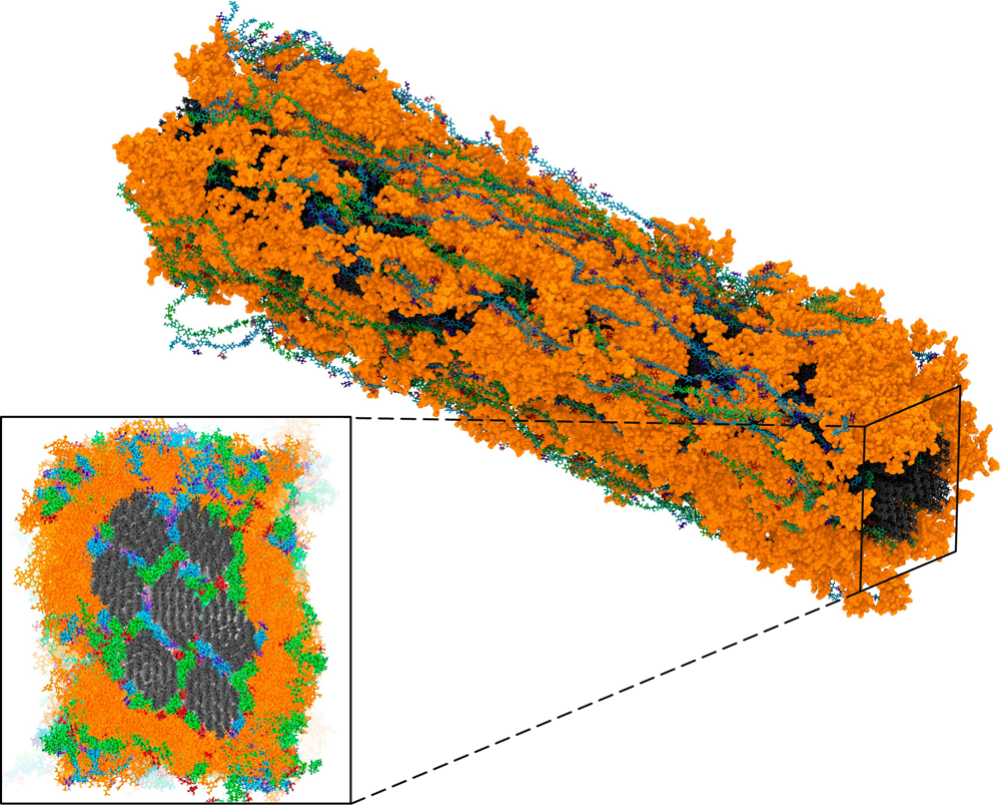
Multicomponent atomistic secondary cell wall for spruce, highlighting the cross-sectional view but omitting the water and calcium ions. The black, green, blue, and orange molecules are cellulose, mannan, xylan, and lignin, respectively. This representation is based on the wet model (898,725 atoms) with 108.4 × 147.5 × 517.9 Å^3^.

The “nolcc” model (899,205 atoms) exhibited a density of 1.33 g cm^–3^, closely resembling that of the “wet” model. The “dry” model (700,992 atoms) was constructed with a density of 1.27 g cm^–3^. Importantly, the densities of all three models remained stable throughout the NPT production runs, indicating system stability.

Simulation trajectories of the “wet” model revealed that lignin aggregates maintained a hydrated state and did not undergo significant coalescence. This observation is consistent with experimental findings in softwoods.^9^ Figure 4 provides a visual representation of the water distribution and key interactions mediated by water molecules within the simulated system.

**Figure 4:**
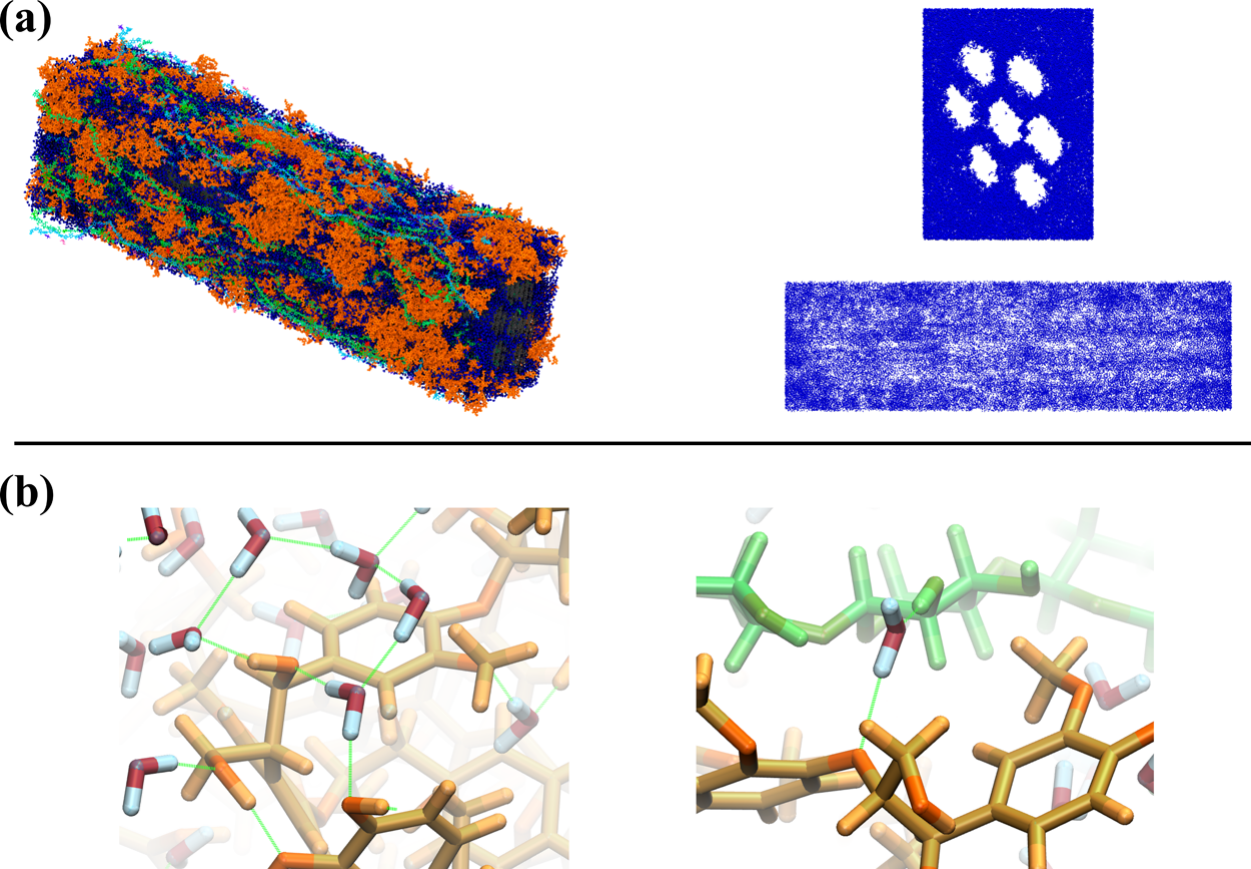
Overview of the water distribution, an essential component of the system, in our models. Panel (a) illustrates the distribution of water molecules in this model from different perspectives, both with and without the visualization of the underlying lignocellulosic matrix. Panel (b) highlights the key role of water in mediating interactions between hemicellulose and lignin, as well as emphasizing the high degree of hydration observed within the lignin domains.

Interestingly, during the equilibration phase, two cellulose microfibrils spontaneously coalesced, overcoming the initial separation imposed by hemicellulose chains. This observation is consistent with recent findings from solid-state NMR and molecular dynamics simulations in poplar hardwood,^40^ which suggest that fused microfibrils may be a prominent feature of cell wall architecture. Utilizing experimental data from poplar, Addison et al.^40^ argue that the coalescence of cellulose fibrils reduces the extent of direct lignin-cellulose contact. While approximately 60–80% of xylan interacts with lignin, a higher proportion of lignin (approximately 80%) is in contact with xylan. These hemicellulose chains, particularly those within the microfibril clusters, may mediate interactions between cellulose chains and influence the coalescence process. Consistent with the findings of Addison et al.^40^ and supported by experimental data from spruce wood,^18^ our model, based on softwood data, also suggests that lignin is primarily localized outside the core of the macrofibril, with hemicellulose potentially acting as a barrier, limiting direct lignin-cellulose interactions.

### Lignin Induces Changes in Hemicellulose Conformation

Our model demonstrated a significant influence of lignin on the conformational distribution of AGX chains adjacent to the cellulose surface. Experimental studies have shown that free xylan residues predominantly adopt a 3-fold conformation (3_1_), whereas xylan chains bound to cellulose typically exhibit a 2-fold conformation (2_1_). In our simulations, hemicellulose conformations were analyzed by plotting histograms of the sum of dihedral angles (φ + ψ) around the glycosidic bond for each monomer pair in the chain. Peaks around 120°, 50°, and 190° correspond to the 2_1_ screw conformation and the right-handed and left-handed 3_1_ screw conformations, respectively.^77^ Notably, the inclusion of lignin in the model favored a shift in the xylan conformational distribution toward the 3_1_ conformation, as illustrated in Figure 5.

**Figure 5:**
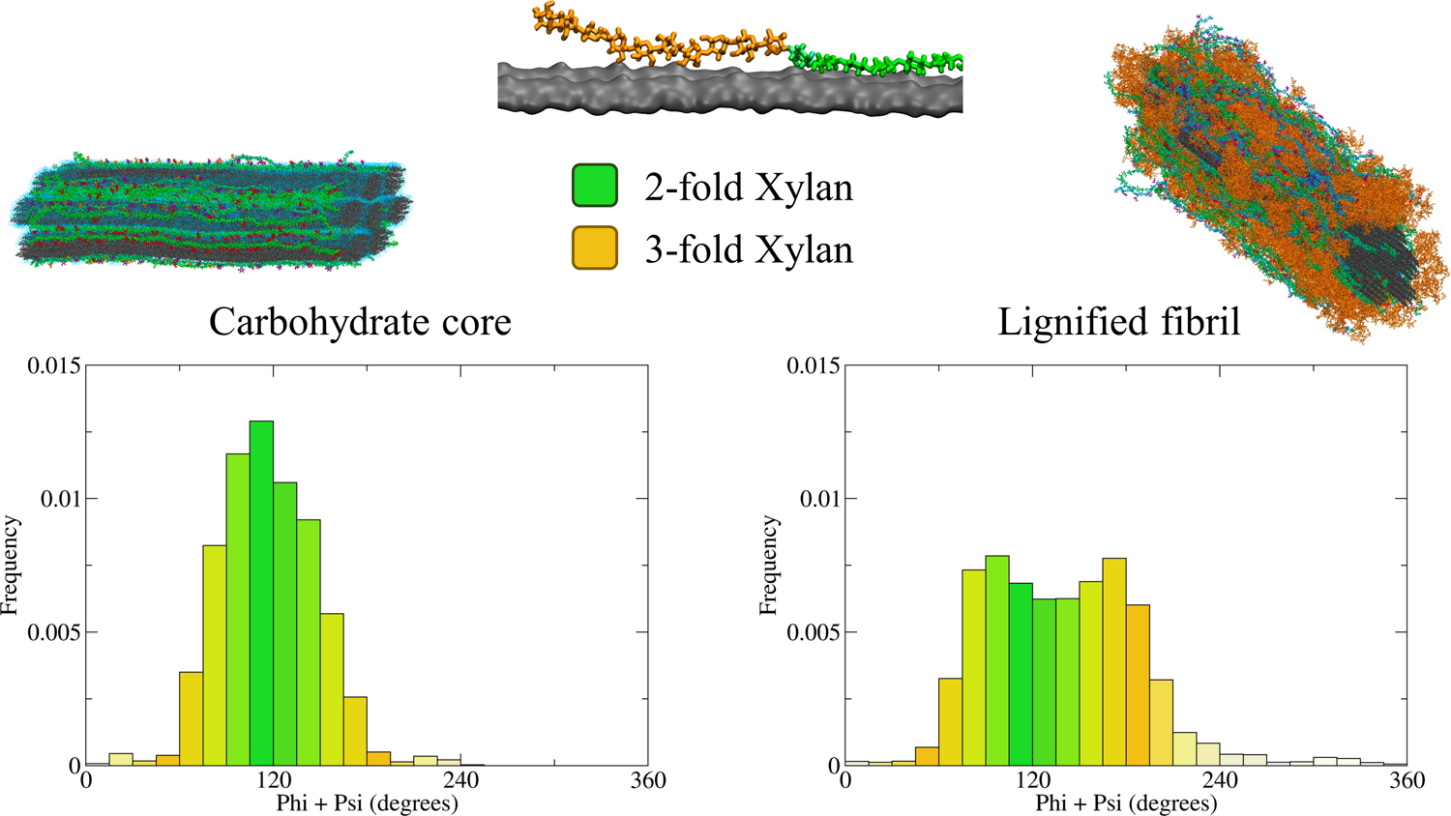
Dihedral angle distributions of hemicellulose chains adjacent to the cellulose crystalline core in the presence and absence of lignin. The results demonstrate that lignin inclusion favors the 3_1_ xylan conformation.

Our findings suggest that lignin formation may promote the 3-fold conformation of xylan in spruce. Previous studies^34^ demonstrated that thermal pretreatment (at 433 K) induces a shift in xylan chains from the hydrophilic to the hydrophobic domains of the cellulose microfibril. Considering that lignin typically occupies the hydrophobic domains outside the cellulose crystalline cluster, these phenolic compounds may interact with xylan chains during the initial stages of lignin deposition, favoring the 3_1_ conformation over the 2_1_ conformation in the context of xylan-cellulose interactions. This effect was particularly pronounced in the presence of covalent lignin-carbohydrate complexes (LCCs). Simulation trajectories revealed that the incorporation of large, covalently linked lignin fragments significantly influenced the attachment of hemicellulose to the cellulose microfibril at specific points, consequently promoting the 3_1_ conformation. This effect proved robust, persisting through subsequent heating and pressurization steps. This computational finding is in agreement with available solid-state NMR data, which show that xylan chains in the predominantly 3_1_ conformation regions lie close to lignin nanodomains.^9,11^ However, a significant fraction of xylan fragments maintained the 2-fold conformation when adsorbed to the microfibril, consistent with experimental observations.^60,74^

While the lignification process induces similar behavior in galactoglucomannan (GGM) chains within the polysaccharide fibrils, the conformational distributions of mannan and xylan chains vary significantly depending on their proximity to cellulose fibrils, as shown in Figure 6. Mannan chains adjacent to cellulose (adsorbed) are predominantly found in the 2_1_ conformation, whereas xylan chains near cellulose exhibit a bimodal distribution, indicating that different segments of AGX chains are found in different conformations. AGX segments in the 3_1_ conformation are mainly those in the extremities of the chains or middle chain straps that detach from the cellulose surface. Conversely, both mannan and xylan chains located far from cellulose (partially adsorbed) primarily adopt the 3_1_ conformation. Further analyses showed that adsorbed chains have a mean hemicellulose-cellulose interchain separation of approximately 5 Å, while partially adsorbed chains exhibit a mean interchain separation of approximately 18 Å; see Figure S3. These conformational differences highlight that mannans exhibit a substantially stronger interaction with cellulose microfibrils compared to arabinoglucuronoxylan (AGX) chains, suggesting a critical role for mannans in softwood cell wall architecture.

**Figure 6:**
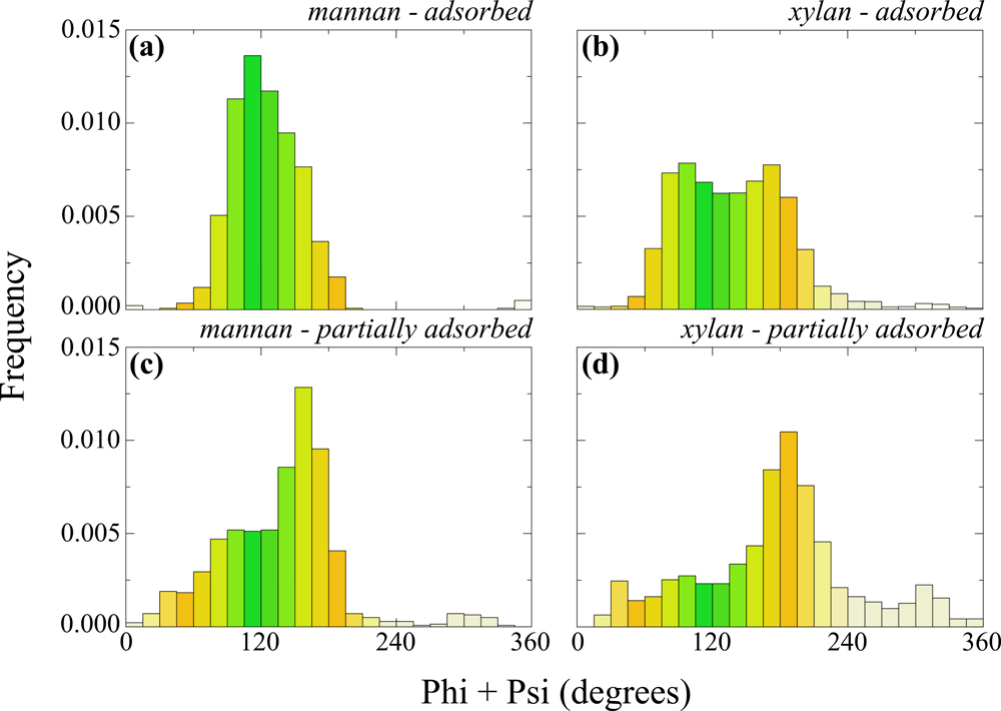
Backbone dihedral angle distributions of hemicellulose, mannan (a, c), and xylan (b, d) chains adjacent to and far from the cellulose crystalline core for lignified fibrils. The results demonstrate that, compared to xylan chains, mannans adsorb more effectively on cellulose as the 2_1_ conformation predominates.

### Interstitial Water Behavior

Water may occupy interstitial regions between the microfibril surface and the adsorbed hemicellulose fraction, mediating the interactions between these carbohydrates.^17,18^ The interstitial water molecules are expected to act differently for xylan–cellulose interfaces and mannan–cellulose interfaces.^18^ Here, we computed the hydrogen-bond survival probability function, *f*_HB_(*t*),^87,88^ to monitor the dynamics of water molecules located at the AGX-cellulose and GGM-cellulose interfaces for each of the cellulose-adsorbed hemicellulose chains. Details of the computations and fitting of the H-bond survival probability functions are provided as Supporting Information. The time integral of *f*_HB_(*t*) provides a rough estimate of the average residence time of the interstitial water molecules (Figure S4). Detailed fitting information is described in the SI.

The average residence times, τ_avg_, of water at the AGX–cellulose interstitial regions are somewhat greater than the corresponding residence times at the GGM–cellulose interfaces (7.6 and 5.8 ns, respectively). However, there is a higher dispersion of τ_avg_ values at AGX–cellulose (2.0–25.9 ns) than at GGM–cellulose interfaces (0.3–11.7 ns), as shown in Tables S2 and S3. These results are consistent with our previous reports^18^ using a simpler hemicellulose–cellulose binding model, although the present residence times span much larger values depending on where the hemicellulose chains are bound on the cellulose surfaces.

Our findings indicate a reduced residence time for water molecules located within the hydrophobic cellulose-hemicellulose interface, relative to those interacting with hydrophilic regions (see Figure S5). Monitoring the two interaction types shown in Figure S5, we determined that water molecules exhibited an approximate 5 ns increase in residence time within the xylan-cellulose interface, particularly for chains adsorbed onto hydrophilic cellulose surfaces. Consistent with the backbone dihedral conformational analysis, the reduced average residence time at the mannan-cellulose interface suggests a stronger adhesive interaction between mannans and the cellulose surface.^12^

### Mechanical Properties of the S2 Models

To assess the mechanical behavior of each equilibrated model, both tensile and compressive tests were conducted by using two distinct strain rates: 10^–5^ (fast) and 10^–7^ fs^–1^ (slow). During these simulations, strain and stress values, Young’s Modulus, and per-atom stress distributions were calculated and analyzed. Below, we describe the results of the tensile and compressive tests in detail.

#### Tensile Ttest

The tensile test simulations, where the simulation box was stretched, exhibited quasi-linear elastic behavior, as illustrated in Figure 7.

**Figure 7:**
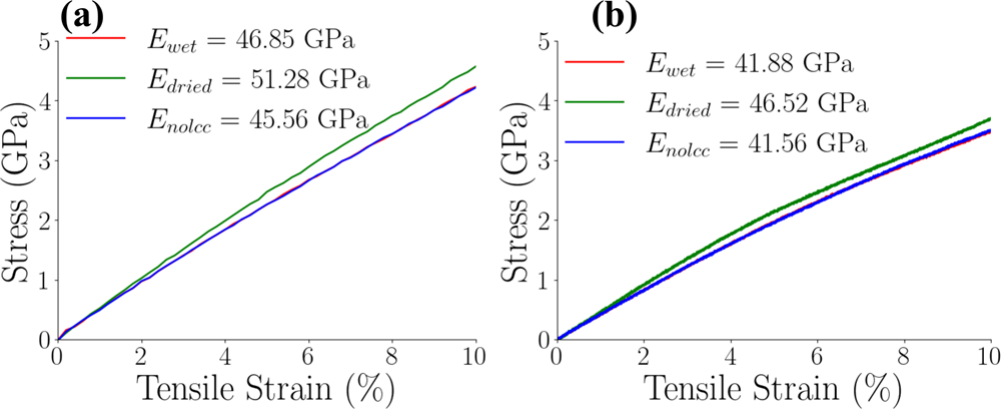
Tensile stress–strain curves for wet, dry, and nolcc models at strain rates of (a) 1 × 10^–5^ fs^–1^ and (b) 1 × 10^–7^ fs^–1^. Young’s Modulus values are indicated in the legend.

The tensile simulations did not exhibit distinct yields or fracture points. This is an inherent limitation of classical MD simulations, where covalent bonds are modeled as idealized springs that can be stretched indefinitely, regardless of the applied force or strain rate. To mitigate this limitation and obtain reliable estimates of Young’s Modulus, only small strain values (ranging from 0.1 to 1%) were considered for the linear regression analysis.

Figure 7 clearly demonstrates a strain rate dependence of Young’s Modulus, with simulations performed at the slower strain rate (10^–7^ fs^–1^) exhibiting lower values. This observation suggests viscoelastic behavior within the simulated cell wall. While the “fast” simulations (10^–5^ fs^–1^) provide valuable insights into the dynamic stress distribution within the model, their high strain rates are not representative of typical experimental conditions. Therefore, the Young’s Modulus values obtained from the ‘slow’ simulations are considered more relevant for comparison with experimental data.

Zhang et al.^37^ reported Young’s Moduli for PCWs ranging from 8 to 40 GPa, with values for the S2L structure reaching approximately 43 GPa. Their MD simulations of the S2 structure yielded Young’s Moduli between 36 and 44 GPa. Gibson^89^ reported PCW moduli of up to 30 GPa, while Deng et al.^90^ reported values of approximately 70 GPa for plant cell walls with a tensile strength of 1 GPa. Specifically for spruce wood, a Young’s Modulus of approximately 30 GPa has been reported.^91^ The Young’s Modulus values obtained in our simulations, as depicted in Figure 7, fall within this range of experimentally and computationally determined values. Similar Young Moduli are also obtained in our compression tests, as shown below (cf. Figure 12). It is important to note that the literature presents a wide range of values for plant cell wall mechanical properties, reflecting the significant variability in experimental techniques and the inherent heterogeneity of plant cell walls.^91–94^ While our simulations provide valuable insights, it is crucial to acknowledge that the mechanical properties of individual constituents, such as cellulose microfibrils (∼130 GPa), hemicelluloses (∼5 GPa), and lignin (∼3 GPa), exhibit a narrower range of variability in comparison to the whole cell wall.^37,89,90^

Interestingly, the “dry” model exhibited a higher Young’s Modulus compared to the “wet” model. This observation aligns with experimental findings demonstrating that both cellulose and wood exhibit increased stiffness at lower moisture content.^18,95–98^ These findings suggest that water molecules act as an internal lubricant within the cell wall, reducing friction forces between microfibrils and facilitating their relative movement.^99–101^

This phenomenon is entangled with the difference in water diffusion between the two models in the NPT ensemble (Figure S6).^102–106^ The water self-diffusion coefficients for the “wet” and ‘dry’ systems were 26.5 × 10^–9^ and 2.2 × 10^–9^ cm^2^/s, respectively (see the SI for computational details). Our results indicate a 12-fold increase in water diffusion within the hydrated model compared to the dried state, with both diffusion coefficients remaining lower than that of bulk water.^107^ This observation echoes Sarkar et al.,^41^ who reported a roughly 10-fold increase in water diffusion from dry to wet states, also below bulk water. Regarding absolute values, the diffusion coefficients obtained from our models were approximately an order of magnitude smaller than those reported by Sarkar et al. at similar hydration levels. This discrepancy is likely a consequence of differences in the way water molecules are distributed in the two model systems. Specifically, our PCW model, with its more uniform hydration distribution within the polymeric matrix, imposes greater constraints on water molecules, thus reducing their displacement compared to Sarkar et al.’s system. In their model, water molecules appear to predominantly surround the polysaccharide bundle at similar hydration levels.

Experimental studies have shown that LCCs contribute significantly to the mechanical properties of plant cell walls.^27^ However, in our simulations, no significant differences in mechanical properties were observed between the “wet” model and the “nolcc” model (which lacks covalent links between lignin and hemicellulose). This discrepancy may be partially attributed to the relatively low density of LCCs in our model. It is possible that a more pronounced effect of LCCs on the mechanical properties would be observed with a higher density of LCCs. However, simulating systems with significantly higher LCC densities poses substantial computational challenges. Moreover, a much slower strain rate could also facilitate the identification of the influence of LCCs on the mechanical properties.

#### Per-Atom Stress Distribution during Tension

To gain insight into the mechanical role of each constituent within the cell wall, the per-atom stress along the *z*-axis (σ_*zz*_) was calculated, and its distribution was visualized using normalized histograms. Given the more pronounced differences observed in the “fast” simulations, this analysis focused on the data obtained at a strain rate of 10^–5^ fs^–1^. Figure 8 presents the normalized histograms of per-atom stress along the *z*-axis for each constituent within the cell wall.

**Figure 8:**
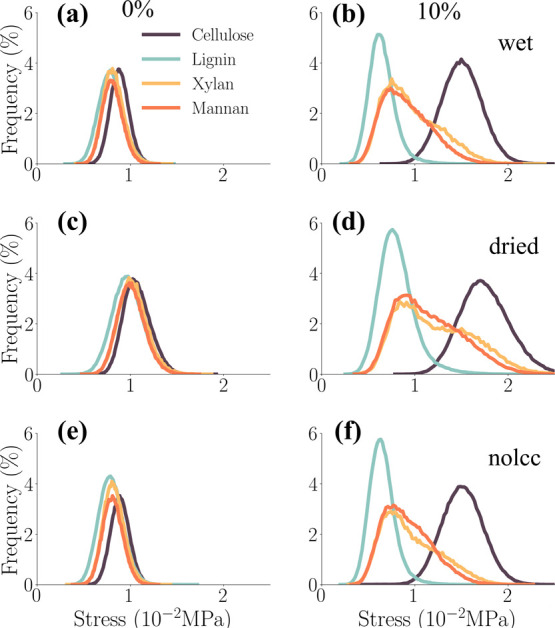
Normalized histograms of per-atom tensile stress σ_*zz*_ obtained at a strain rate of 10^–5^ fs^–1^ for wet (top), dried (center), and nolcc (bottom) models at 0% (left) and 10% (right) strain. The constituents of the biocomposite are displayed in different colors.

As evident in Figure 8, cellulose microfibrils experience the highest stress levels during tensile loading, as indicated by the right-most black curves at 10% strain. This observation is further supported by Figure 9, which highlights the stress distribution within the cellulose microfibrils in red. Hemicellulose exhibits intermediate stress loads, while lignin experiences the lowest.

**Figure 9:**
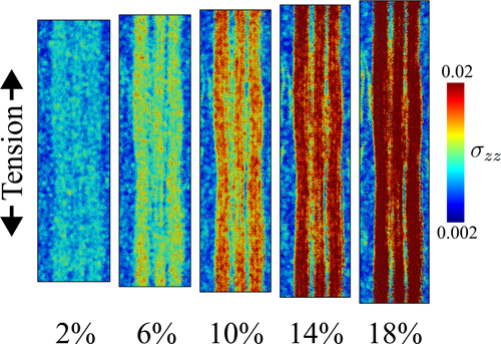
Wet model being subjected to tensile stress from 2 to 18% strain. Per-atom stresses (MPa) are plotted in a jet color map, where the axial stress loading is greater when the color is red and lower when it is blue.

The “dry” model consistently demonstrated higher stress levels across all constituents, as evidenced by a rightward shift in the stress distribution curves compared to the ‘wet’ and ‘nolcc’ models. This observation aligns with previous findings,^27^ which demonstrated that hemicellulose exhibits higher mechanical properties than lignin in bamboo fibrils. We hypothesize that this increased stress within the “dry” model arises from stronger interactions between cellulose fibrils in the absence of significant water-mediated lubrication. Furthermore, we propose that lignin, through strong van der Waals interactions with the cellulose surface, may contribute to the overall stiffness of the system, while hemicellulose-lignin interactions, predominantly driven by hydrogen bonds, may exhibit more flexibility.

Intriguingly, the stress distribution for lignin (light blue lines, Figure 8) decreases slightly with increased strain for all three models. This behavior likely occurs because the lignins, initially added to the system as globules, can unfold during stretching, thus relieving internal stress. The initial globular entangled shape may therefore display higher stresses than a less globular stretched lignin. Furthermore, the increased stress of cellulose may also imply that other components not connected to it play a less important role in bearing the tensile loads. It is not uncommon that the fibers in a matrix+fibers composite take the stage in a tensile test, moving the matrix's importance to the side.

The hemicellulose stress distributions, as depicted by the orange and yellow curves in Figure 8, exhibit a unique bimodal character. This intriguing distribution suggests a heterogeneous stress environment within the hemicellulose population. We hypothesize that this bimodality arises from variations in hemicellulose-microfibril interactions. Hemicellulose chains in close proximity to the cellulose surface, likely bound through extensive hydrogen bonding, experience stress levels that closely mirror those of the adjacent cellulose microfibrils. In contrast, hemicellulose chains with weaker or more distant interactions with the cellulose surface, potentially located further from the microfibril surface, experience stress levels more akin to those surrounding the lignin and aqueous regions. This observation supports the notion that hemicellulose plays a crucial role in mediating mechanical interactions within the cell wall matrix.^60,74^

To further investigate the computed stress distribution within the hemicellulose population, hemicellulose chains were categorized on the basis of their proximity to the cellulose surface: “fully adsorbed” (adjacent to the cellulose surface) and “partially adsorbed” (further apart from the cellulose surface). The distance distribution between the backbone atoms of each hemicellulose residue (xylose and mannose units) and the nearest atoms within the cellulose microfibrils (Figure S3) was calculated using the MolecularMinimumDistances.jl package.^108^

In the unloaded state (0% strain), the stress distributions for the fully and partially adsorbed hemicellulose chains exhibited minimal differences. Therefore, to effectively differentiate between these two populations, the analysis focused on the stress distributions at the maximum applied strain (10%), as depicted in Figure 10.

**Figure 10:**
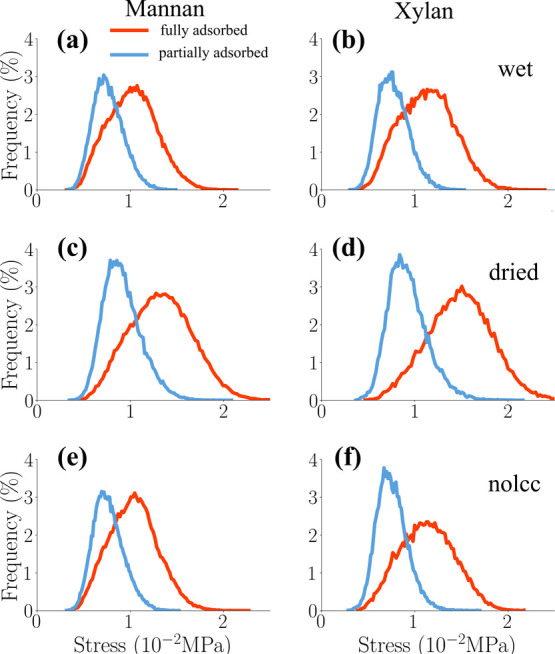
Normalized histograms of per-atom tensile stress σ_*zz*_ along the *z*-axis for mannan and xylan of wet (top), dry (center), and nolcc (bottom) models at 10% strain. Mannans and Xylans are classified into fully and partially adsorbed to the surface of the surface of the cellulose fibrils.

The analysis revealed that both xylan and mannan chains classified as adsorbed (i.e., those located in close proximity to the cellulose surface) exhibited significantly higher stress levels compared with those partially adsorbed. This observation supports the hypothesis that hemicellulose chains in close proximity to the cellulose surface experience higher stress levels because of their direct involvement in transmitting mechanical forces within the cell wall. Figure 11 provides a representation of adsorbed and partially adsorbed hemicellulose chains under tensile loading.

**Figure 11:**
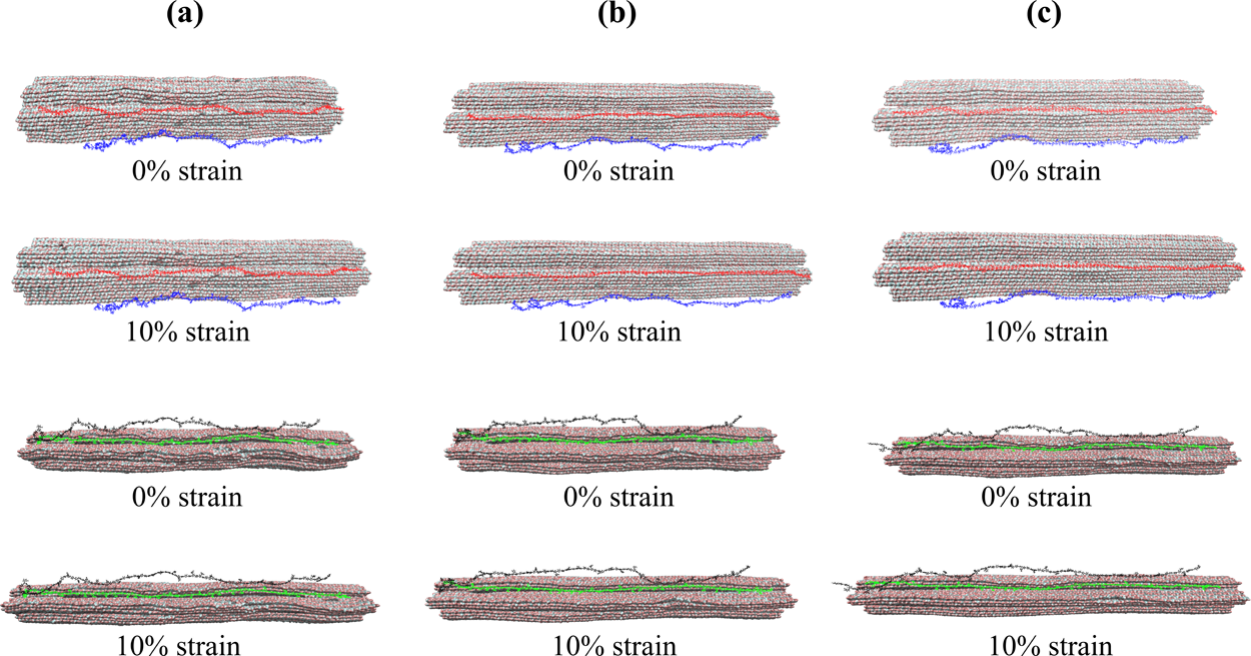
Visual representation of the “dry” (a), “wet” (b), and “nolcc” (c) models under tensile loading. The two top rows depict the distribution of mannan chains within each model, with partially adsorbed mannans shown in blue (AN33) and fully adsorbed mannans in red (AN10). The two bottom rows depict the distribution of xylan chains, with partially adsorbed xylans shown in black (XY21) and adsorbed xylans in green (XY8). These visualizations illustrate the spatial distribution of hemicellulose chains within each model and their relative proximity to the cellulose microfibrils under tensile loading at 0 and 10% strain, as indicated.

#### Compressive Test

Similarly to the tensile tests, the compressive stress–strain curves exhibited an initial quasi-linear elastic region, followed by a yield point. The yield point occurred at approximately 6% strain in the “fast” simulations and around 2% strain in the “slow” simulations. Subsequent to the yield point, strain softening behavior was observed in all models. Notably, the “dry” model exhibited strain hardening during the “slow” compression simulation, as evidenced by the green curve in Figure 12b.

**Figure 12:**
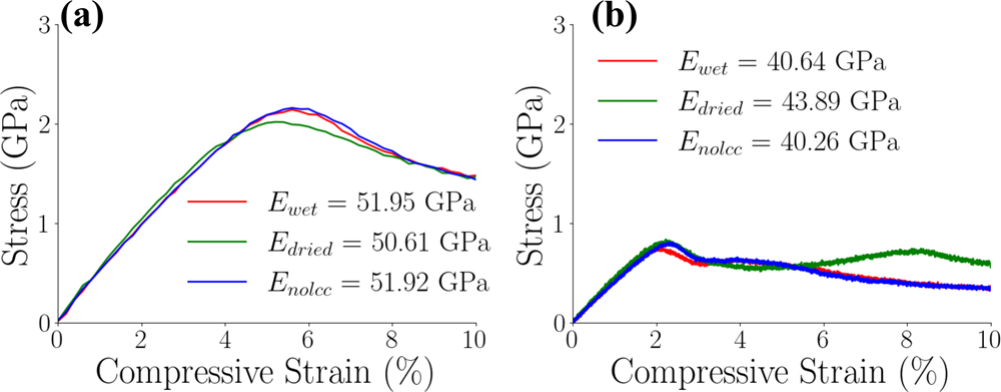
Compressive stress–Strain curves for wet, dried, and nolcc models. (a) Simulations with a strain rate of 10^–5^ fs^–1^. (b) Simulations with a strain rate of 10^–7^ fs^–1^. The legends display the Young’s Modulus values for each curve.

The observed compressive behavior of our models exhibits characteristics similar to those of ductile polymers (thermoplastics),^92,109–112^ featuring an initial linear elastic region followed by a yield point and subsequent strain softening. Consistent with the tensile tests, Figure 12 shows a dependence of the Young’s Modulus on the strain rate, with simulations performed at the slower strain rate exhibiting lower values. Furthermore, the “dry” model consistently displayed higher Young’s Modulus values compared to the “wet” model, mirroring the trends observed in tensile simulations. Similarly to the findings in the tensile tests, no significant differences in mechanical properties were observed between the “wet” and “nolcc” models under compressive loading.

#### Per-Atom Stress Distribution during Compression

Figure 13 presents the distribution of per-atom stresses along the *z*-axis for each constituent during compressive loading. To effectively differentiate the stress distributions within each constituent, the analysis focused on the stress state at 6% strain, a point near the yield point in the ‘fast’ compression simulations. In contrast to the unique bimodal distributions observed in the tensile tests, both mannan and xylan exhibited more Gaussian-like stress distributions during compression.

**Figure 13:**
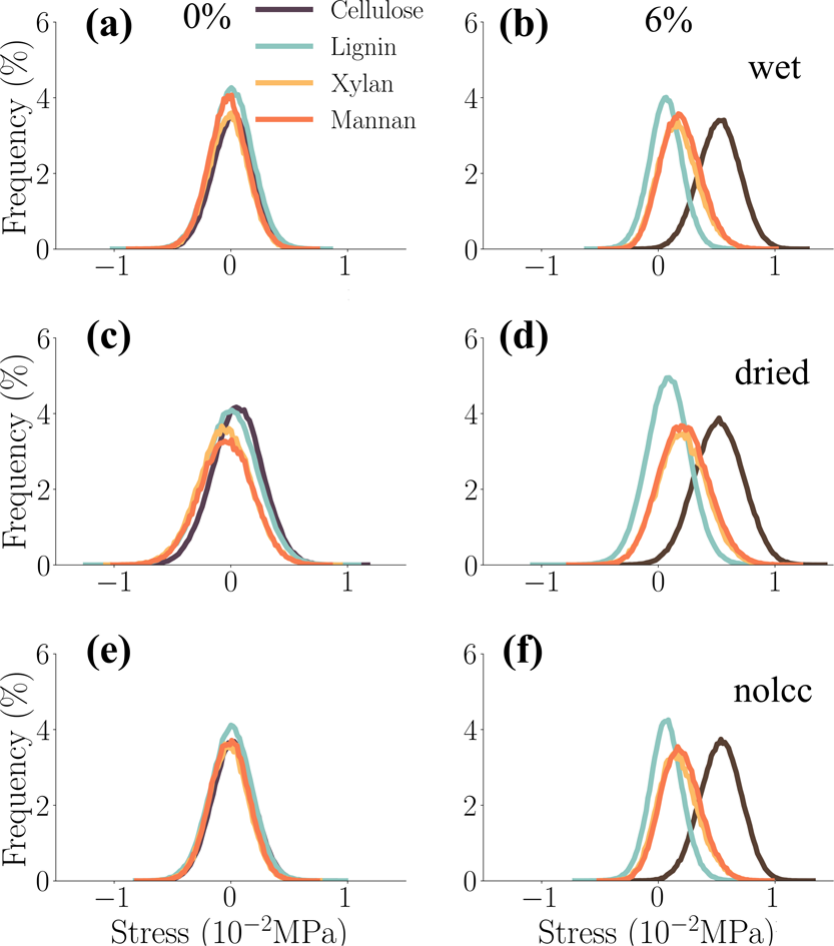
Normalized histograms of per-atom compressive stress σ_*zz*_ along the *z*-axis for wet (top), dried (center), and nolcc (bottom) models at 0% (left) and 6% (right) strain. The constituents of the biocomposite are displayed in different colors.

As observed in tensile simulations, cellulose microfibrils exhibited the highest stress levels during compressive loading, particularly at 4 and 6% strain, as evident in the red profiles in Figure 14. Conversely, lignin experienced the lowest stress levels.

**Figure 14:**
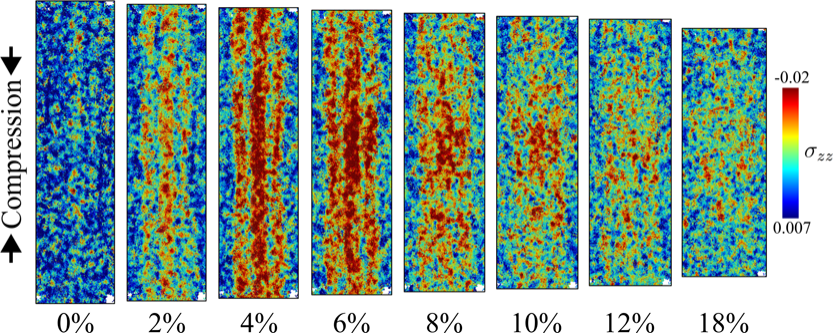
Wet model subjected to compression stress from 0 to 18% strain. Per-atom stresses (MPa) are plotted in a jet color map, where the axial stress loading is greater when the color is red and lower when it is blue.

We also computed the compression stress distributions for the two hemicellulose subpopulations: “adsorbed” and “partially adsorbed”, as previously defined, as shown in Figure 15).

**Figure 15:**
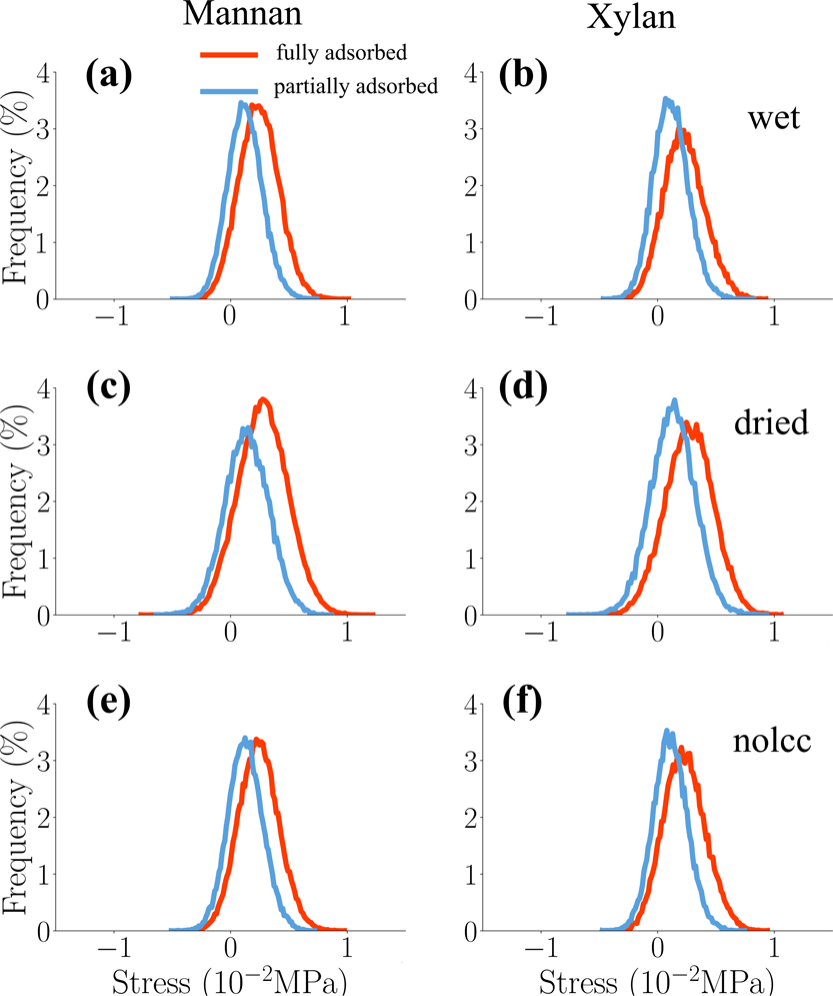
Normalized histograms of per-atom compressive stress σ_*zz*_ along the *z*-axis for mannan and xylan in the “wet” (top), “dry” (center), and “nolcc” (bottom) models at 6% strain. Hemicellulose chains are categorized as “adsorbed” (in close proximity to the cellulose surface) and “partially adsorbed” (more distant from the cellulose surface).

In contrast to the tensile loading scenario, no significant differences in mechanical behavior were observed between fully and partially adsorbed hemicellulose groups under compression. Figure 16 visually distinguishes between these two hemicellulose populations. The average distance from the cellulose surface was approximately 5 Å for adsorbed hemicellulose residues and 20 Å for partially adsorbed residues. In the ‘dry’ model, the maximum distance between the hemicellulose chains and the cellulose surface was observed to be approximately 15 Å.

**Figure 16:**
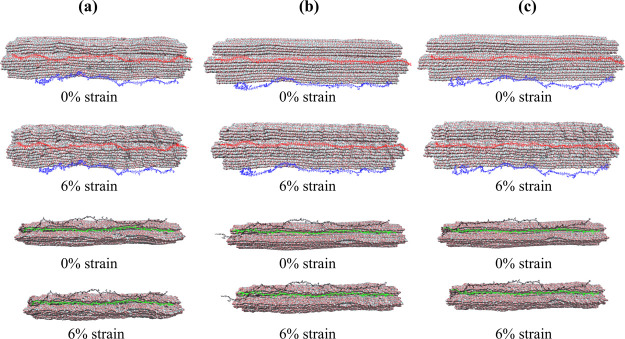
Visual representation of the “dry” (a), “wet” (b), and “nolcc” (c) models under compressive loading. The two top rows depict the distribution of mannan chains within each model, with adsorbed mannans shown in red (AN10) and partially adsorbed mannans shown in blue (AN33). The two bottom rows depict the distribution of xylan chains, with adsorbed xylans colored green (XY8) and partially adsorbed xylans colored black (XY21). These visualizations illustrate the spatial distribution of hemicellulose chains within each model and their relative proximity to the cellulose microfibrils under compressive loading.

While lignin is often cited as a key contributor to the compressive strength of the cell wall,^4^ our results suggest that its primary role may not be to directly withstand high compressive stresses. Instead, the lignin matrix appears to play a critical role in stress dissipation within the cell wall. As observed in Figure 14, lignin contributes to a more uniform distribution of compressive stress across the cellulose fibrils. This observation extends to the tensile simulations, where lignin and its associated LCCs appear to facilitate the distribution of tensile stress to hemicellulose chains not directly bound to the cellulose surface.

#### Buckling

During slow compression simulations, localized buckling is observed in the microfibrils, characterized by sudden changes in shape (deformation) when the strain exceeds a critical value (approximately 2%). These buckling events, visually evident in Figure 17 as localized distortions, likely contribute to the observed strain softening behavior, manifested as fluctuations in the stress–strain curve (Figure 12b) beyond the yield point.

**Figure 17:**
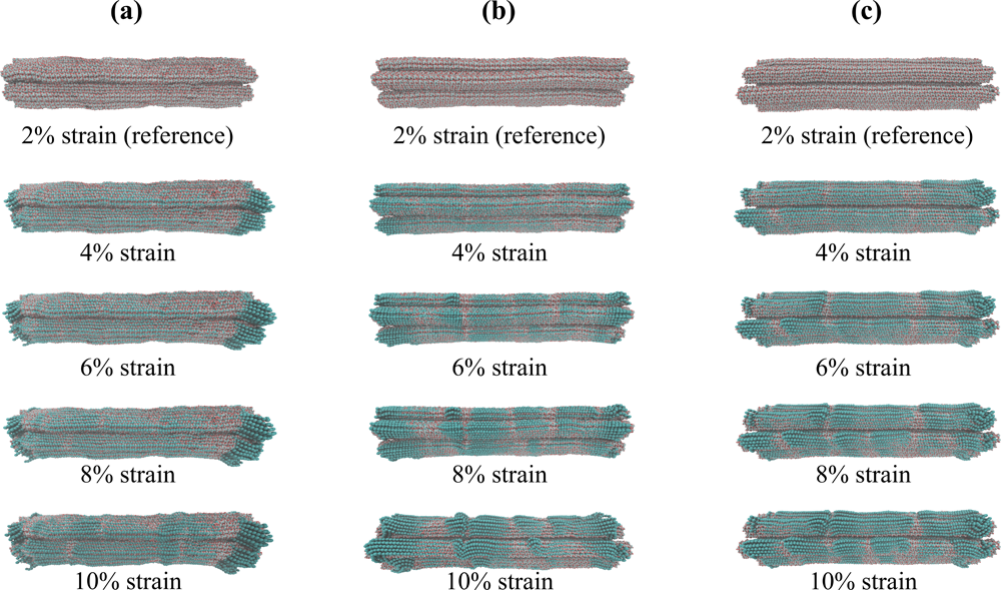
Visualization of the “dry” (a), “wet” (b), and “nolcc” (c) models under compressive loading. Buckling, characterized by localized deformations, are evident in the cellulose fibrils of the ‘wet’ and ‘nolcc’ models at strains exceeding 2% (gray). The terminal regions of the fibrils, particularly in the ‘dry’ model, exhibit a more disordered configuration. For reference, the 2% strain state is highlighted in gray, alongside the other strain values (blue). Note that the models are not drawn to scale.

## Conclusions

In this study, we investigated the mechanical behavior of spruce wood secondary cell walls’ S2 layer by constructing a multicomponent atomistic model informed by experimental data. The model incorporated key structural features, including cellulose microfibrils interconnected via periodic boundary conditions, a hemicellulose matrix composed of xylan and galactoglucomannan, a lignin matrix representative of softwood composition, and lignin-carbohydrate complexes. This multicomponent model accurately reflects the hierarchical organization observed in native secondary cell walls.

From a structural point of view, the constructed models exhibited realistic densities within the expected range (1.3–1.5 g cm^–3^) and displayed stable structural integrity throughout the simulations. Conformational analysis revealed a dynamic interplay between hemicellulose conformations, with 2-fold conformations prevalent near cellulose surfaces and 3-fold conformations observed in regions more distant from the microfibrils. Water molecules permeated the matrix, mediating noncovalent interactions between lignin and carbohydrates and likely acting as a “molecular lubricant”,^113,114^ facilitating relative movement between polymer chains during deformation. Water molecules also intermediate interactions between cellulose and hemicellulose, playing the role of “structural” water, with some of the average residence times approaching 30 ns.

Investigations of the mechanical behavior of our models showed that the wet, dried, and nolcc models exhibit quasi-linear elastic behavior under tensile loading. In contrast, compressive stress–strain curves displayed initial linear elasticity, followed by strain softening and hardening typical of polymer behaviors, with localized buckling observed during slow compression. Stress distribution analyses indicated that cellulose microfibrils bear most of the load in both tension and compression. While lignin exhibited relatively low stress levels, it likely plays a role in stress dissipation within the cell wall matrix. Analysis of hemicellulose stress distributions revealed that those chains in close proximity to the cellulose surface (adsorbed) exhibited higher stress levels under tension, suggesting a significant role in load transfer. In contrast, no significant differences in stress levels were observed between the fully and partially adsorbed hemicellulose chains under compression.

While the present study did not reveal a significant impact of covalent lignin-carbohydrate bonds under the simulated loading conditions, it is anticipated that LCCs would play a more prominent role in scenarios involving higher LCC density, larger deformations, or more extreme loading conditions, such as those leading to cell wall detachment or microfibril slippage. This study provides valuable insights into the mechanical mechanisms and interactions within spruce softwood’s secondary cell walls, underscoring the critical roles of each component in maintaining structural integrity.

## Supplementary Material



## Notes

Steps of the simulation protocols to model the S2 system; RMSD for constructed models and per-residue backbone distance to cellulose fibril structure for fully and partially adsorbed hemicellulose; hydrogen-bond survival probability functions for interstitial water molecules; mean squared displacement for water molecules within the polymeric matrix; system files and scripts are available at https://zenodo.org/records/15103315: distance.jl: Julia script used to compute the hemicellulose-cellulose distances.diffusion.jl: Julia script to compute the MSD and diffusion coefficient.time.jl: Julia script to compute the survival probability functions for interstitial water; and system files: .psf, .pdb, and other parameters for each system (PDF)
