# Supplementary material for: Unraveling the Mechanical Behavior of Softwood Secondary Cell Walls through Atomistic Simulations
Source: Biomacromolecules. 2025 May 27;26(6):3395–409. doi: 10.1021/acs.biomac.4c01806 (PMC12153074; doi:10.1021/acs.biomac.4c01806)
Supplement: Supplementary file 1 [file bm4c01806_si_001.pdf]

Supporting Information: Unraveling the Mechanical Behavior of Softwood Secondary Cell Walls through Atomistic Simulations

Lucas N. Trentin,[†,‡] Amadeus C. S. Alcântara,[¶,‡] Carlos G. T. Batista,[†,‡] and Munir S. Skaf[*,†,‡]

†*Institute of Chemistry, Campinas, SP 13084-862, Brazil*

‡*Center for Computing in Engineering & Sciences (CCES), Campinas, SP 13083-861, Brazil*

¶*Department of Computational Mechanics, School of Mechanical Engineering, Campinas, SP 13083-860, Brazil*

E-mail: skaf@unicamp.br

- The following system files and scripts are available at

  `https://zenodo.org/records/15103315` ; DOI:10.5281/zenodo.15103315

  distance.jl: julia script used to compute the hemicellulose-cellulose distances.

  diffusion.jl: julia script to compute the MSD and diffusion coefficient.

  time.jl: julia script to compute the time of interstitial water.

  System files: .psf, .pdb, and others parameters for each system.

- **System Specifications**: cellulose, xylan (AGX), mannan (GGM) and lignin are the main building blocks of the three cell wall systems of plants. For practical purposes in PDB/PSF formats, sugar chains were identified without explicit chain labeling but designated according to their positions.

  The **cellulose** chains were labeled as follows: A5 A6 A7 A11 A12 A13 A14 A15 A16 A19 A20 A21 A22 A23 A24 A28 A29 A30 B5 B6 B7 B11 B12 B13 B14 B15 B16 B19 B20 B21 B22 B23 B24 B28 B29 B30 C5 C6 C7 C11 C12 C13 C14 C15 C16 C19 C20 C21 C22 C23 C24 C28 C29 C30 D5 D6 D7 D11 D12 D13 D14 D15 D16 D19 D20 D21 D22 D23 D24 D28 D29 D30 E5 E6 E7 E11 E12 E13 E14 E15 E16 E19 E20 E21 E22 E23 E24 E28 E29 E30 F5 F6 F7 F11 F12 F13 F14 F15 F16 F19 F20 F21 F22 F23 F24 F28 F29 F30 G5 G6 G7 G11 G12 G13 G14 G15 G16 G19 G20 G21 G22 G23 G24 G28 G29 G30. The external cellulose chains are 14, 23, 30, 29, 28, 19, 11, 12, 5, 6, 7, and 16. Each microfibril was codified with alphabetical letters (A–G). As an example, the chain D15 is part of the microfibril D.

  The **xylans** (AGXs) were labeled:

  Close cellulose surface: XY1 XY2 XY3 XY4 XY5 XY6 XY7 XY8 XY9 XY10 XY11 XY12 XY13 XY14 XY15 XY16

  Distant from cellulose macrofibril: XY21 XY22 XA17 XB17 XC17 XA18 XB18 XC18 XA19 XB19 XC19 XA20 XB20 XC20

Crosslinked with lignins on LCCs: XA17 XB17 XC17 XA18 XB18 XC18 XA19 XB19 XC19 XA20 XB20 XC20.

The **Mannans** (GGMs) were labeled:

Close to cellulose: AN1 AN2 AN3 AN4 AN5 AN6 AN7 AN8 AN9 AN10 AN11 AN12 AN13 AN14 AN15 AN16 AN17 AN18 AN19 AN20 AN21 AN22 AN23 AN24

Between the macrofibril core and the outside matrix: AN31 AN32 AN33

Distant from cellulose surface: MA25 MB25 MC25 MA26 MB26 MC26 MA27 MB27 MC27 MA28 MB28 MC28 MA29 MB29 MC29 MA30 MB30 MC30

Participating in lignin crosslinking: MA25 MB25 MC25 MA26 MB26 MC26 MA27 MB27 MC27 MA28 MB28 MC28 MA29 MB29 MC29 MA30 MB30 MC30

Accessible (40% acetylated) mannans, 4:1:0.3 ratio): AN1 AN2 AN3 AN4 AN5 AN6 AN7 AN8 AN9 AN10 AN11 AN12 AN13 AN14 AN15 AN16 AN31 AN32 MA25 MB25 MC25 MA26 MB26 MC26 MA27 MB27 MC27 MA28 MB28 MC28

Recalcitrant (10% acetylated) mannans, 3:1:1.2 ratio: AN17 AN18 AN19 AN20 AN21 AN22 AN23 AN24 AN33 MA29 MB29 MC29 MA30 MB30 MC30.

The **lignins** chains were labeled:

Solvated lignins: J1 J2 J3 J4 J5 J6 J7 J8 J9 J10 J11 J13 J14 J17 J20 J22 J26 J28 J34 J40 J42 J48 J49 J51 J52 J53 J55 J57 J60 J65 J69 J71 J73 J74 J75 J76 J77 J78 J79 J82 J83 J84 J88 J89 J90 J91 J95 J96 K1 K2 K3 K4 K5 K6 K7 K8 K9 K10 K11 K13 K14 K17 K20 K22 K26 K28 K34 K40 K42 K48 K49 K51 K52 K53 K55 K57 K60 K65 K69 K71 K73 K74 K75 K76 K77 K78 K79 K82 K83 K84 K88 K89 K90 K91 K95 K96 L1 L2 L3 L4 L5 L6 L7 L8 L9 L10 L11 L13 L14 L17 L20 L22 L26 L28 L34 L40 L42 L48 L49 L51 L52 L53 L55 L57 L60 L65 L69 L71 L73 L74 L75 L76 L77 L78 L79 L82 L83 L84 L88 L89 L90 L91 L95 L96

Lignins closer to the macrofibril core: L12A L15A L19A L23A L25A L27A L29A

L32A L44A L45A L46A L56A L64A L66A L68A L70A L85A L87A L92A L98A L12B L15B L19B L23B L25B L27B L29B L32B L44B L45B L46B L56B L64B L66B L68B L70B L85B L87B L92B L98B L12C L15C L19C L23C L25C L27C L29C L32C L44C L45C L46C L56C L64C L66C L68C L70C L85C L87C L92C L98C

Lignins involved in crosslinking with hemicelluloses: J4 J16 J18 J21 J24 J30 J31 J35 J36 J37 J33 J38 J39 J41 J43 J47 J50 J54 J58 J59 J61 J62 J63 J67 J72 J80 J81 J86 J93 J94 J97 J99 K4 K16 K18 K21 K24 K30 K31 K35 K36 K37 K33 K38 K39 K41 K43 K47 K50 K54 K58 K59 K61 K62 K63 K67 K72 K80 K81 K86 K93 K94 K97 K99 L4 L16 L18 L21 L24 L30 L31 L35 L36 L37 L33 L38 L39 L41 L43 L47 L50 L54 L58 L59 L61 L62 L63 L67 L72 L80 L81 L86 L93 L94 L97 L99

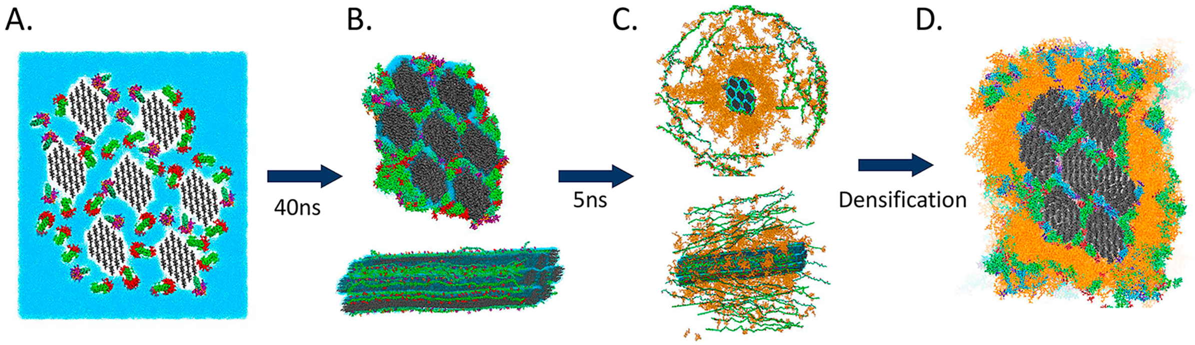

Figure S1: Building protocol applied up to densification of the system. (A) Shows the initial setup with some AGXs and GGMs close to the macrofibril core. The compact carbohydrate core (B) is surrounded by lignins and leftover chains of hemicellulose, (D) leading to the densification process. In this work, the hemicellulose chains close to the core are labeled as fully absorbed and the chains that were added with the lignin matrix is called partially absorbed (i.e. on the cellulose macrofibril core).

Table S1: Simulation protocol used to build the final structure for the plant cell wall model, showing relevant parameters for each step.

Stage	Duration (ps)	Temperature (K)	Pressure (bar)	Constrains
**1**	200	300	1	no
**2**	50	300	6	no
**3**	50	300	10	no
**4**	50	300	15	no
**5**	50	300	20	no
**6**	50	300	25	no
**7**	50	300	35	no
**8**	50	300	50	no
**9**	50	300	80	no
**10**	50	300	150	no
**11**	50	300	250	no
**12**	50	300	400	no

continued on next page

Table S1 – continued from previous page

Stage	Duration (ps)	Temperature (K)	Pressure (bar)	Constrains
**13**	50	300	600	no
**14**	50	300	850	no
**15**	50	300	1100	no
**16**	50	300	1500	no
**17**	50	300	1800	no
**18**	50	300	2100	no
**19**	50	300	2400	no
**20**	50	300	2700	no
**21**	50	300	3000	no
**22**	25	800	-	yes
**23**	50	300	4000	yes
**24**	25	800	-	yes
**25**	50	300	5000	yes
**26**	25	800	-	yes
**27**	50	300	6000	yes
**28**	25	800	-	yes
**29**	50	300	7000	yes
**30**	25	800	-	yes
**31**	50	300	8000	yes
**32**	25	800	-	yes
**33**	50	300	9000	yes
**34**	25	800	-	yes
**35**	50	300	10000	yes
**36**	25	800	-	yes

continued on next page

Table S1 – continued from previous page

Stage	Duration (ps)	Temperature (K)	Pressure (bar)	Constrains
**37**	50	300	10000	yes
**38**	25	800	-	yes
**39**	50	300	10000	yes
**40**	25	800	-	yes
**41**	50	300	10000	yes
**42**	25	800	-	yes
**43**	50	300	2500	no
**44**	50	300	500	no
**45**	50	300	50	no
**46**	700	300	1	no

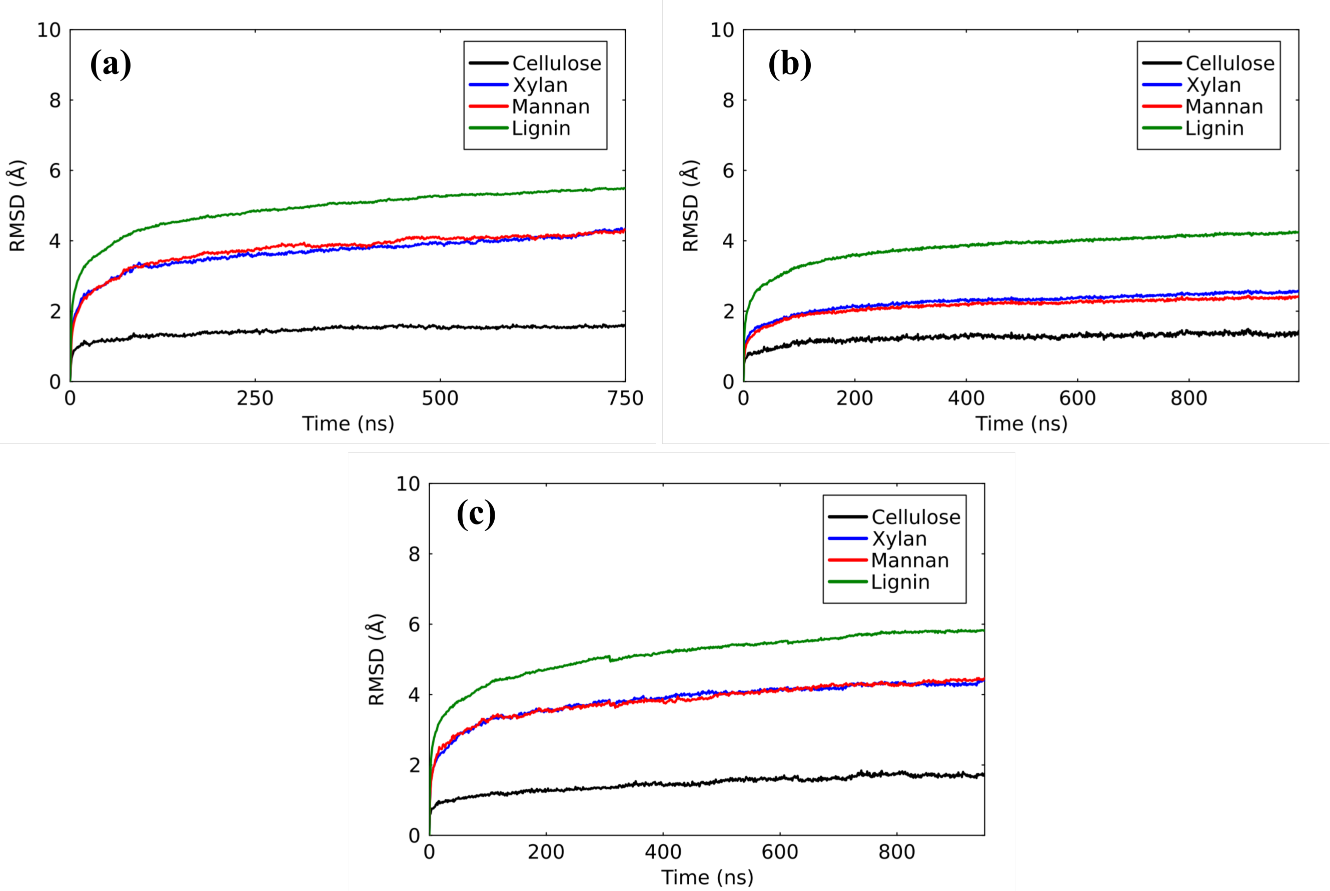

Figure S2: RMSD of the constituents of the (a) wet, (b) dry, and (c) nolcc models based on the initial structures.

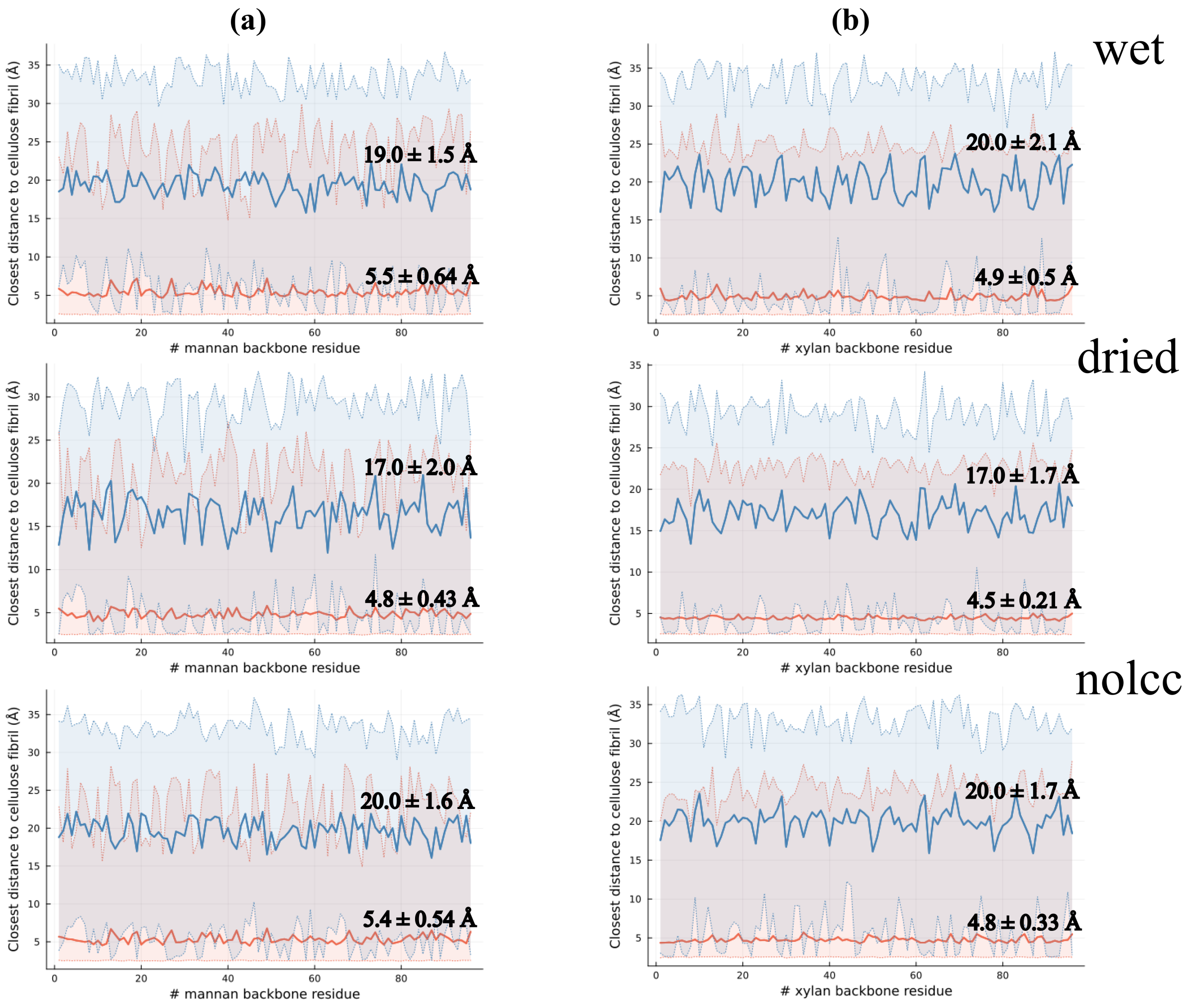

Figure S3: Per-residue backbone distance to cellulose fibril for (a) mannan and (b) xylan. The xylan and mannan chains classified as partially and fully adsorbed are colored blue and orange, respectively. The bold line represents the average distance of the residue along the last 100 ns of equilibration. The orange shaded area is limited by dotted lines, illustrating the minimum and maximum distance reached by each residue during these 100 ns.

Interstitial water: Water molecules making hydrogen bonds to cellulose and hemicellulose simultaneously at a given time were classified as interstitial water at the instant of time. H-bonding was stabilished according to the geometric criteria $r_{O..O} < 3.5$ Å, $r_{H..O} < 2.6$ Å, and $\angle_{OH..O} < 30°$ [8,9]. We focused on cellulose chains located on the surface of each microfibril and the hemicellulose backbone close to this surface, since water molecules are expected to mediate cellulose-hemicellulose interactions. The first two and last two residues of the hemicellulose chain were excluded to avoid high mobility residues. The H-bond survival

probability function, $f_{\mathrm{HB}}(t)$, allowed us to quantify the time lapse during which each water molecule acted as an H-bond donor or acceptor between these carbohydrate chains [9,10]:

$$f_{\mathrm{HB}}(t) = \frac{\langle \eta(0)\eta(t) \rangle}{\langle \eta^2 \rangle}$$

where the characteristic function $\eta(t) = 1$ if a water molecule is identified as interstitial (i.e., simultaneously H-bonded to cellulose and hemicellulose) at time t and zero otherwise.

Excluding the very early time behavior, the $f_{\mathrm{HB}}(t)$ time-correlation can be well-fitted by a bi-exponential function:

$$f_{\mathrm{HB}}(t) \approx A_1\, e^{-t/\tau_1} + A_2\, e^{-t/\tau_2} \; .$$

The best bi-exponential fitting parameters, within time window $0.2 < \mathrm{t} < 300$ ns, for xylan-cellulose and mannan-cellulose interstitial regions are shown in **Table S2** and **Table S3**, respectively. A rough estimate for the average residence time of water at interstitial sites is obtained from the time integral of $f_{\mathrm{HB}}(t)$: $\tau_{\mathrm{avg}} \approx \mathrm{A}_1\ \tau_1 + \mathrm{A}_2\ \tau_2$ [9].

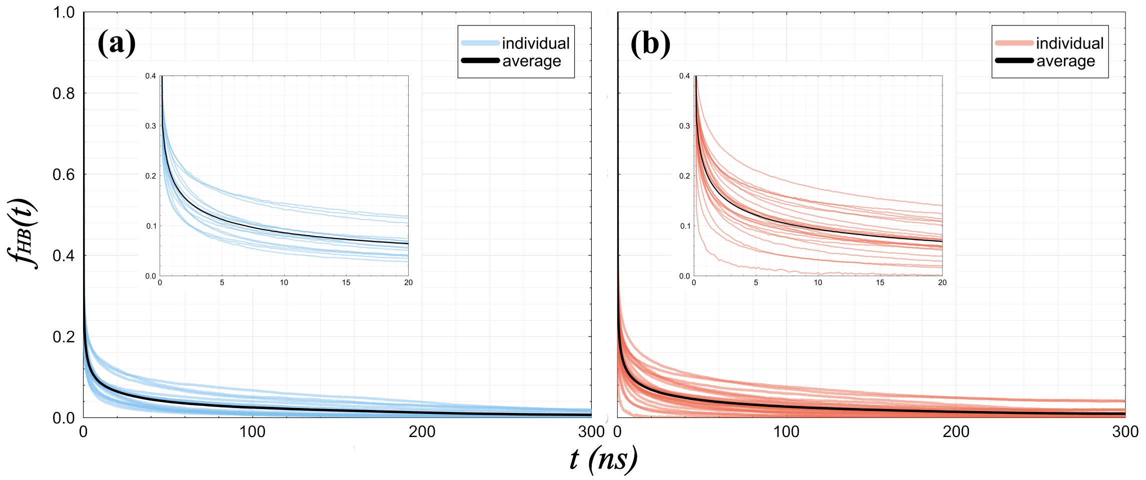

Figure S4: Survival probability function for interstitial water at (a) xylan–cellulose and (b) mannan–cellulose interfaces. The thin lines correspond to each xylan or mannan adjacent to cellulose. The thick lines stand for the averages. Bi-exponential fits to these data were carried out for the 0.2–300 ns time interval of each individual curve. The inserts highlight the 0–20 ns time window.

Table S2: Bi-exponential best-fitting parameters to the $f_{\mathrm{HB}}(t)$ functions depicted in Figure S4 within the 0.2 – 300 ns time interval. The average residence time of interstitial water, τ_{avg}, and the coefficient of determination, R^2, are also shown.

**Chain**	A_1	τ_1	A_2	τ_2	R^2	τ_{avg}
XY1	0.135	38.5	–	–	0.938	5.2
XY2	0.162	5.11	0.127	197	0.998	25.9
XY3	0.135	12.8	0.107	204	0.995	23.5
XY4	0.146	2.06	0.0601	42.2	0.992	2.8
XY5	0.199	2.03	0.0847	36.9	0.994	3.5
XY6	0.150	13.30	–	–	0.951	2.0
XY7	0.158	2.27	0.0681	77.0	0.994	5.6
XY8	0.197	1.92	0.0516	36.6	0.992	2.3
XY9	0.214	2.23	0.0603	48.7	0.991	3.4
XY10	0.224	2.26	0.109	30.6	0.996	3.8
XY11	0.166	7.30	0.0940	152	0.990	15.6
XY12	0.155	3.03	0.0746	92.6	0.996	7.4
XY13	0.139	24.5	–	–	0.942	3.4
XY14	0.160	2.69	0.0785	95.9	0.995	8.0
XY15	0.176	3.36	0.0820	83.5	0.995	7.4
XY16	0.164	1.81	0.0617	36.0	0.994	2.5

Table S3: Exponential fitting of correlation function by time (ns) for each mannan chain close to the macrofibril core. The integral represents the average of water population from both residence time.

**Chain**	A_1	τ_1	A_2	τ_2	R^2	τ_{avg}
AN1	0.230	3.00	0.0773	43.6	0.994	4.1
AN2	0.212	3.52	0.0962	86.9	0.997	9.1

continued on next page

Table S3 – continued from previous page

**Chain**	A_1	τ_1	A_2	τ_2	R^2	τ_{avg}
AN3	0.184	3.00	0.112	99.2	0.995	11.7
AN4	0.217	3.36	0.0810	86.5	0.996	7.7
AN5	0.176	3.61	0.0802	68.2	0.996	6.1
AN6	0.142	2.28	0.0326	47.2	0.991	1.9
AN7	0.175	18.3	–	–	0.951	3.2
AN8	0.190	1.90	0.0784	23.1	0.995	2.2
AN9	0.192	2.67	0.0918	57.4	0.988	5.8
AN10	0.210	1.81	0.0660	27.0	0.993	2.2
AN11	0.168	2.04	0.0746	33.4	0.992	2.8
AN12	0.171	4.24	0.119	73.1	0.997	9.5
AN13	0.164	2.59	0.0661	83.8	0.994	6.0
AN14	0.213	1.22	0.0603	16.8	0.995	1.3
AN15	0.201	3.48	0.118	49.7	0.997	6.6
AN16	0.167	3.80	0.0912	72.3	0.995	7.2
AN17	0.187	2.20	0.0794	46.0	0.994	4.1
AN18	0.147	3.63	0.0671	109.0	0.991	7.8
AN19	0.129	0.500	0.0432	5.12	0.994	0.3
AN20	0.178	2.96	0.0711	60.1	0.989	4.8
AN21	0.185	3.32	0.136	60.9	0.996	8.9
AN22	0.171	5.35	0.118	83.2	0.997	10.7
AN23	0.177	2.78	0.108	88.4	0.996	10.0
AN24	0.187	1.46	0.0868	45.2	0.998	4.2

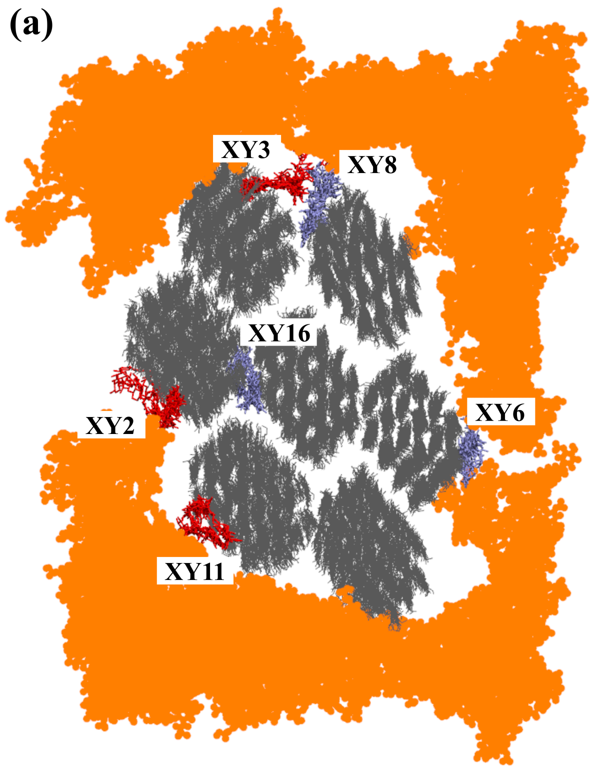

Figure S5: Cross-sectional view of the polymeric matrix of 'wet' system. The (a) xylans chains on the hydrophilic region of cellulose (red) present highest resident time than the chains on hydrophobic face (blue). We found the same behavior on (b) mannan–cellulose interface.

Self-diffusion coefficient: The diffusion coefficients of water for the three models (wet, dried, and nolcc) were estimated using the Einstein-Smoluchowski expression [1,2]:

$$6\,D\,t = \lim_{t\to\infty} \left\langle [\mathbf{r}_i(t) - \mathbf{r}_i(0)]^2 \right\rangle$$

For this purpose, the mean squared displacement (MSD) was calculated for the hydrated and dehydrated systems, unwrapped using the qwrap plugin[1], following the algorithm of Bullerjahn, von Bülow, and Hummer [3] . Given the TIP3P water model used to build the system, the expected values are on the order of 5.9–6.2 $\times 10^{-5}$ cm^2/s [4]. To validate our calculations, we used a 25 Å $\times$ 25 Å $\times$ 25 Å water box containing 522 TIP3P water molecules at 1.0 atm and 298.15 K. After the minimization and equilibra-

[1]https://github.com/jhenin/qwrap

tion steps, a 200 ns NpT production run was performed using a 2.0 fs timestep, with frames captured every 1 ps. The diffusion coefficient obtained was $5.04 \times 10^{-5}\,\text{cm}^2/\text{s}$, consistent with the values reported in simulations using NAMD [5,6]. The diffusion coefficients for the hydrated and dehydrated systems were $26.5 \times 10^{-9}\,\text{cm}^2/\text{s}$ and $2.21 \times 10^{-9}\,\text{cm}^2/\text{s}$, respectively, calculated using trajectories after 200 ns of equilibration, sampled every 0.2 ns. These values were lower than those reported by Sarkar et al. (2023). Yet, they display the same trend. The water diffusion in the hydrated system was approximately 10 times higher than in the dehydrated system, with both cases exhibiting lower values compared with bulk water [7]. The organization of the dry matrix suggests an influence on the absolute differences between our results and Sarkar's study. The more homogeneous hydration distribution in our models systems compared to Sarkar et al.'s may have constrained water molecules within the polymeric matrix, reducing the displacement of structural water.

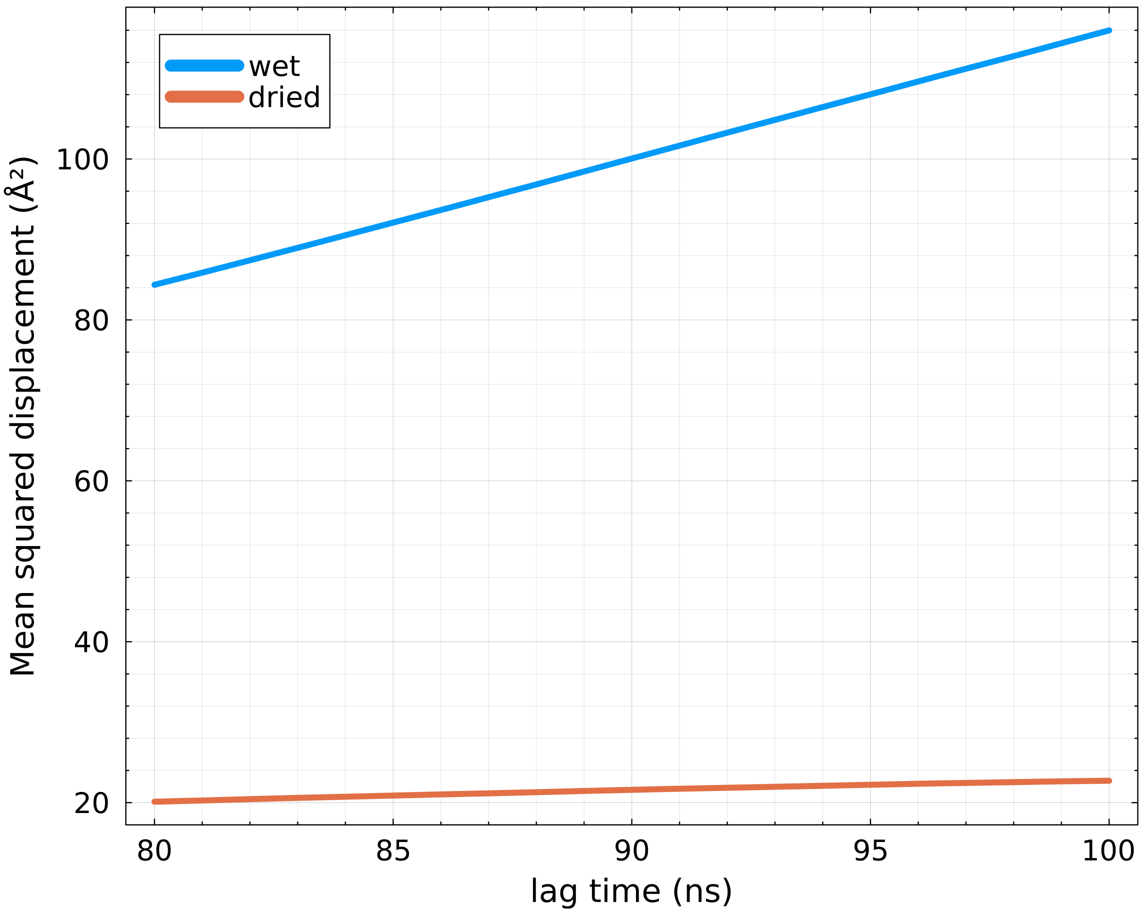

Figure S6: Linear range used to fit the self-diffusion coefficient of 'wet' (blue) and 'dried' (orange) models.

REFERENCES

(**1**) A. Einstein, *Annalen der Physik* **1905**, 322, 549–560.

(**2**) M. Von Smoluchowski, *Annalen der Physik* **1906**, 326, 756–780.

(**3**) J. T. Bullerjahn, S. Von Bülow, G. Hummer, *The Journal of Chemical Physics* **2020**, 153, 024116.

(**4**) P. Mark, L. Nilsson, *J. Phys. Chem. A* **2001**, 105, 9954.

(**5**) J. T. Bullerjahn, S. Von Bülow, M. Heidari, J. Hénin, G. Hummer, *J. Chem. Theory Comput.* **2023**, 19, 3406.

(**6**) M. Kulke, J. V. Vermaas, *J. Chem. Theory Comput.* **2022**, 18, 6161.

(**7**) D. Sarkar, L. Bu, J. E. Jakes, J. K. Zieba, I. D. Kaufman, M. F. Crowley, P. N. Ciesielski, J. V. Vermaas, *The Cell Surface* **2023**, 9, 100105.

(**8**) W. L. Jorgensen, J. Chandrasekhar, J. D. Madura, R. W. Impey, M. L. Klein, *The Journal of Chemical Physics* **1983**, 79, 926.

(**9**) M. H. H. Pomata, M. T. Sonoda, M. S. Skaf, M. D. Elola, *J. Phys. Chem. B* **2009**, 113, 12999.

(**10**) M. T. Sonoda, M. S. Skaf, *J. Phys. Chem. B* **2007**, 111, 11948.
